# SPDL1 Overexpression Is Associated With the 18F-FDG PET/CT Metabolic Parameters, Prognosis, and Progression of Esophageal Cancer

**DOI:** 10.3389/fgene.2022.798020

**Published:** 2022-05-18

**Authors:** Hua-Song Liu, Qiang Guo, Heng Yang, Min Zeng, Li-Qiang Xu, Qun-Xian Zhang, Hua Liu, Jia-Long Guo, Jun Zhang

**Affiliations:** Department of Cardiothoracic Surgery, Taihe Hospital, Hubei University of Medicine, Shiyan, China

**Keywords:** spindle apparatus coiled-coil protein 1, esophageal cancer, prognosis, biomarker, positron emission tomography/computed tomography

## Abstract

Esophageal cancer (ESCA) is one of the common malignant tumors. The roles and signaling mechanisms of spindle apparatus coiled-coil protein 1 (SPDL1) in ESCA progression have not been reported previously. Therefore, the expression levels and potential clinical roles of *SPDL1* were investigated using data from multiple databases and tissue samples of 53 ESCA patients who underwent 18F-FDG positron emission tomography (PET)/computed tomography (CT) before therapy. The signaling mechanisms of SPDL1 involved in ESCA progression were investigated via bioinformatics analysis. The effects of SPDL1 on the growth and migration of ESCA cells were investigated using CCK-8, Edu, and transwell assays. *SPDL1* was upregulated in ESCA tissues. Increased *SPDL1* expression was associated with age, grade, drinking history, cancer stage, lymph node metastasis, TP53 mutation, and poor prognosis in patients with ESCA. *SPDL1* overexpression was significantly correlated with SUVmax, SUVmean, and TLG of PET/CT. *SPDL1* silencing inhibited cell proliferation, migration, and invasion. *SPDL1* was significantly enriched in cell cycle, spliceosome, DNA replication, and other processes. The hub genes of a constructed protein–protein interaction network included CDK1, BUB1, CCNB1, BUB1B, CCNA2, CDC20, MAD2L1, AURKB, NDC80, and PLK1, which were related to *SPDL1* expression. The findings of this study suggest that SPDL1 may serve as a biomarker of ESCA prognosis.

## 1 Introduction

Esophageal cancer (ESCA) is one of the most common cancers worldwide, including China, with the ninth-highest incidence and sixth-highest mortality worldwide (Chen et al., 2016; Bray et al., 2018). At present, due to the lack of target molecules for early diagnosis, drug treatment, and evaluation of prognosis of ESCA, the five-year survival rate of cancer patients remains low (Zarean et al., 2018; Jingu et al., 2019; Lin et al., 2019). Therefore, new biomarkers are sought after to improve the diagnosis rate and to find new targeted therapies to improve the overall survival (OS) of ESCA patients.

Under physiological conditions, the mitotic spindle can regulate the microtubule cytoskeleton. However, under pathological conditions, abnormal regulation of microtubule cytoskeleton leads to genomic instability, which is associated with cancer progression (Qin et al., 2017). For example, Polo-like kinase 1 (PLK1) is a key kinase that regulates mitosis. The PLK1-specific inhibitor BI2536 can activate spindle assembly checkpoint (SAC) in non-small cell lung cancer cells. Excessive activation of SAC results in cell death (Choi et al., 2015). Mitotic arrest defective protein 2 (*MAD2*) is a key gene that regulates mitosis. *MAD2* expression level is correlated with age, lymph node metastasis, and survival time of small-cell lung cancer patients (Wu et al., 2018). Spindle apparatus coiled-coil protein 1 (*SPDL1*), also known as CCDC99, located at 5q35.1, encodes a protein containing a helical domain, which plays an important role in mitotic spindle formation and chromosome segregation (Barisic et al., 2010; Kodama et al., 2019). The myocardium-associated transcription factor B (MRTFB) can suppress the invasion and migration of colorectal cancer (CRC) cells. Disrupting *SPDL1* expression in CRC cells significantly increases the invasion and migration of CRC cells. In the intestinal tract of mice, the knockdown of *MRTFB* inhibits *SPDL1* expression. Decreased *SPDL1* expression is associated with a low survival rate of CRC. Regulation of melanoma cell adhesion molecule and *SPDL1* promotes the development of xenografts in nude mice (Kodama et al., 2019). This indicates that *SPDL1* functions as a tumor suppressor gene in CRC development. However, the role of SPDL1 in ESCA development has not been reported. Therefore, we evaluated *SPDL1* expression in ESCA tissues via UALCAN, The Cancer Genome Atlas (TCGA), and TIMER database analyses, and used clinical tissues in the present study. We aimed to determine the relationship between *SPDL1* expression and clinical values in patients with ESCA and determine the signaling mechanisms associated with SPDL1 that are involved in the progression of ESCA.

## 2 Materials and Methods

### 2.1 UALCAN Database

The correlation between *SPDL1* expression in ESCA tissues and the clinicopathological features (race, sex, age, drinking history, body weight, histological subtype, smoking history, cancer stage, tumor grade, and TP53 mutation status) of ESCA patients were investigated using the UALCAN (http://ualcan.path.uab.edu) database (Chandrashekar et al., 2017).

### 2.2 The Cancer Genome Atlas and Genotype-Tissue Expression Databases

The gene expression data of pan-cancer cases were downloaded from the TCGA (https://portal.gdc.cancer.gov/) and GTEx (https://gtexportal.org/home/datasets) databases and were analyzed to determine *SPDL1* expression levels. In total, 11,093 tissue profiles of cancer patients were downloaded from the TCGA database, 4,124 tissue profiles were downloaded from the GTEx database, and 15,776 tissue profiles were downloaded from the XENA-TCGA- GTEx database. The data from TCGA and GTEx databases were corrected, normalized, processed for fold-changes, and merged. In total, 171 cases of TCGA ESCA HTSeq-FPKM, including 11 cases of normal esophagus tissues and 160 cases of ESCA tissues, were obtained, and 8 esophagus tissues and 8 ESCA tissues were derived from the same patients. *SPDL1* expression in ESCA tissues was analyzed for TCGA-derived data. The clinical data of 183 patients with ESCA obtained from the TCGA database were analyzed to determine the prognosis of ESCA patients after collating the clinical data.

### 2.3 Clinical ESCA Tissue Samples

Cancer tissues and adjacent tissues were collected from 53 ESCA patients, and *SPDL1* expression in cancer tissues was determined via immunohistochemistry (IHC). This study was approved by the ethics committee of Taihe Hospital. The clinical data of patients, including diagnosis age, gender, T stage, and positron emission tomography (PET)/computed tomography (CT) index, were collected and applied to analyze the *SPDL1* expression level in clinical roles.

### 2.4 Immunohistochemistry

ESCA paraffin sections were deparaffinized. The sections were incubated with 3% H_2_O_2_ at room temperature for 5 min, after which antigen retrieval was performed. The samples were blocked with 10% goat serum and incubated with 1:100 SPDL1 (Proteintech, China) antibody working solution at 4°C overnight. Subsequently, an appropriate amount of biotin-labeled secondary antibody working solution was added following incubation at 37°C. After dolichos biflorus agglutinin staining, counterstaining, dehydration, transparency, mounting and photographing, and other processes, the *SPDL1* expression level in ESCA tissues was determined.

### 2.5 Genes Co-Expressed With *SPDL1*

R (version: 3.6.1) was used to filter genes co-expressed with *SPDL1* in 160 ESCA tissues. Genes associated with *p* < 0.001 and Pearson’s coefficient (r > 0.4 or < −0.4) were considered to be strongly co-expressed with *SPDL1* (Guo et al., 2020). Pearson’s coefficient indicated the association between the two genes to demonstrate the roles of SPDL1.

### 2.6 Gene Ontology, Kyoto Encyclopedia of Genes and Genomes, and Gene Set Enrichment Analysis

The biological functions and signaling pathways of genes co-expressed with *SPDL1* were investigated using GO and KEGG. The 160 ESCA cases were assigned to high-SPDL1 and low-SPDL1 expression groups according to the median value of *SDPL1* expression. The effect of *SPDL1* expression level on each gene was investigated using GSEA, and each analysis was performed 1,000 times (Guo et al., 2021; Zhang et al., 2021).

### 2.7 Protein–Protein Interaction Network in the Search Tool for the Retrieval of Interacting Genes

The STRING (https://string-db.org/) database was applied to investigate the PPI of multiple genes. Genes co-expressed with *SPDL1* were evaluated using the STRING database to establish the PPI network using a combined score >0.7. The top 10 highly connected co-expressed genes were identified using CytoHubba plug-in in Cytoscape 3.6.1 and defined as hub genes. The TCGA and GTEx databases were analyzed using the Gene Expression Profiling Interactive Analysis (GEPIA) database to determine the expression levels of hub genes.

### 2.8 Cell Culture and Transfection

EC109 cells were cultured in Dulbecco’s modified Eagle medium (DMEM) (HyClone, China), and cells showing optimal growth were plated in 6-well plates and cultured for 24 h. *SPDL1* small interfering RNA (siRNA) or microRNA (miRNA) mimics/inhibitors were transfected using Lipofectamine 3000 (Invitrogen, Waltham, MA, USA). SPDL1 mRNA and protein expression levels in *SPDL1* siRNA-transfected (si-SPDL1) and negative control siRNA-transfected (si-NC) groups were determined via quantitative reverse transcription-polymerase chain reaction (qRT-PCR) and western blotting (WB), respectively. *SPDL1* siRNA was purchased from GenePharma (Shanghai, China), and the sense sequence is GGG​AGA​AGU​UUA​UCG​AUU​ATT, and the antisense sequence is UAA​UCG​AUA​AAC​UUC​UCC​CTT that interfere with SPDL1 expression. Total RNA and proteins were extracted according to standard procedures, and PCR and WB were performed (Liu et al., 2020). PCR primers were purchased from Genecopoeia (HQP105394) in China, and anti-SPDL1 antibodies were purchased from Proteintech (China).

### 2.9 Cell Proliferation

Transfected EC109 cells were digested using trypsin and resuspended in 96-well plates. Each group was dispensed in three duplicate wells. Subsequently, 10 µL/well of CCK-8 was added daily, following incubation for 1 h, and the optical density at 450 nm was determined for each well. Transfected EC109 cells (5 × 10^3^ cells/well) were seeded in the 96-well plate. The plates were incubated after addition of pre-warmed Edu working solution (Genecopoeia, China) and an equal volume of medium. EC109 cells were fixed in 4% neutral paraformaldehyde for 15–30 min. Subsequently, the fixative was removed and cells were washed, followed by Edu detection, DNA counterstaining, imaging, and analysis. The experiment was repeated thrice.

### 2.10 Cell Migration and Invasion

Transwell chambers were coated with 1:10 diluted Matrigel, and the transwell chambers were placed in a 24-well plate. Transfected EC109 cells were suspended in serum-free DMEM medium and seeded in transwell chambers. DMEM (500 μL) and 10% fetal bovine serum were added to the bottom of the chamber following incubation at 37°C. After 24 h, the chamber was removed, fixed with 4% neutral paraformaldehyde, stained with crystal violet, and the cells were photographed and counted. The experiment was repeated thrice.

### 2.11 Statistical Analysis

*SPDL1* expression in cancer tissues and the effects of SPDL1 on proliferation, migration, and invasion of ESCA cells were evaluated using the *t*-test. The values of *SPDL1* expression level in ESCA patient diagnosis and prognosis were analyzed via the receiver operating characteristic (ROC) curve and survival analysis. The chi-square test was applied to investigate the relationship between *SPDL1* expression in clinical tissues and clinicopathological characteristics. Values of *p* < 0.05 were considered significant.

## 3 Results

### 3.1 *SPDL1* Was Abnormally Expressed in Pan-Cancer Tissues

**FIGURE 1:**
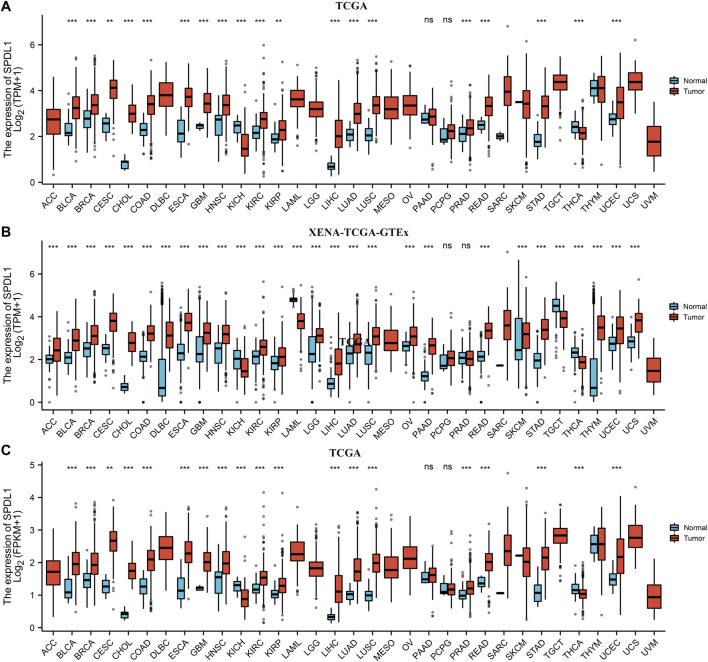
Pan-cancer data from multiple databases showed that *SPDL1* was abnormally expressed in pan-cancer tissues. **(A)** TPM-type data from the TCGA database. **(B)** TPM-type data from the TCGA + GTEx databases. **(C)** FPKM-type data from the TCGA database. SPDL1, spindle apparatus coiled-coil protein 1; TCGA, The Cancer Genome Atlas; GTEx, Genotype-Tissue Expression; TPM, transcripts per million; FPKM, fragments per kilobase per million; NS, not significant; *, *p* < 0.05; **, *p* < 0.01; ***, *p* < 0.001.

*SPDL1* was abnormally expressed in pan-cancer tissues based on data obtained from the TCGA and XENA-TCGA-GTEx databases (Figure 1). The pan-cancer data from the TCGA database were sorted which showed that *SPDL1* was abnormally expressed in several matched cancer tissues (Supplementary Figure S1). *SPDL1* was upregulated in bladder cancer, breast cancer, cholangiocarcinoma, ESCA, squamous cell cancer in the head and neck region, kidney renal clear cell carcinoma, and other tissues, but was downregulated in kidney chromophobe tissues (Supplementary Figure S1).

### 3.2 *SPDL1* Was Upregulated in ESCA

**FIGURE 2:**
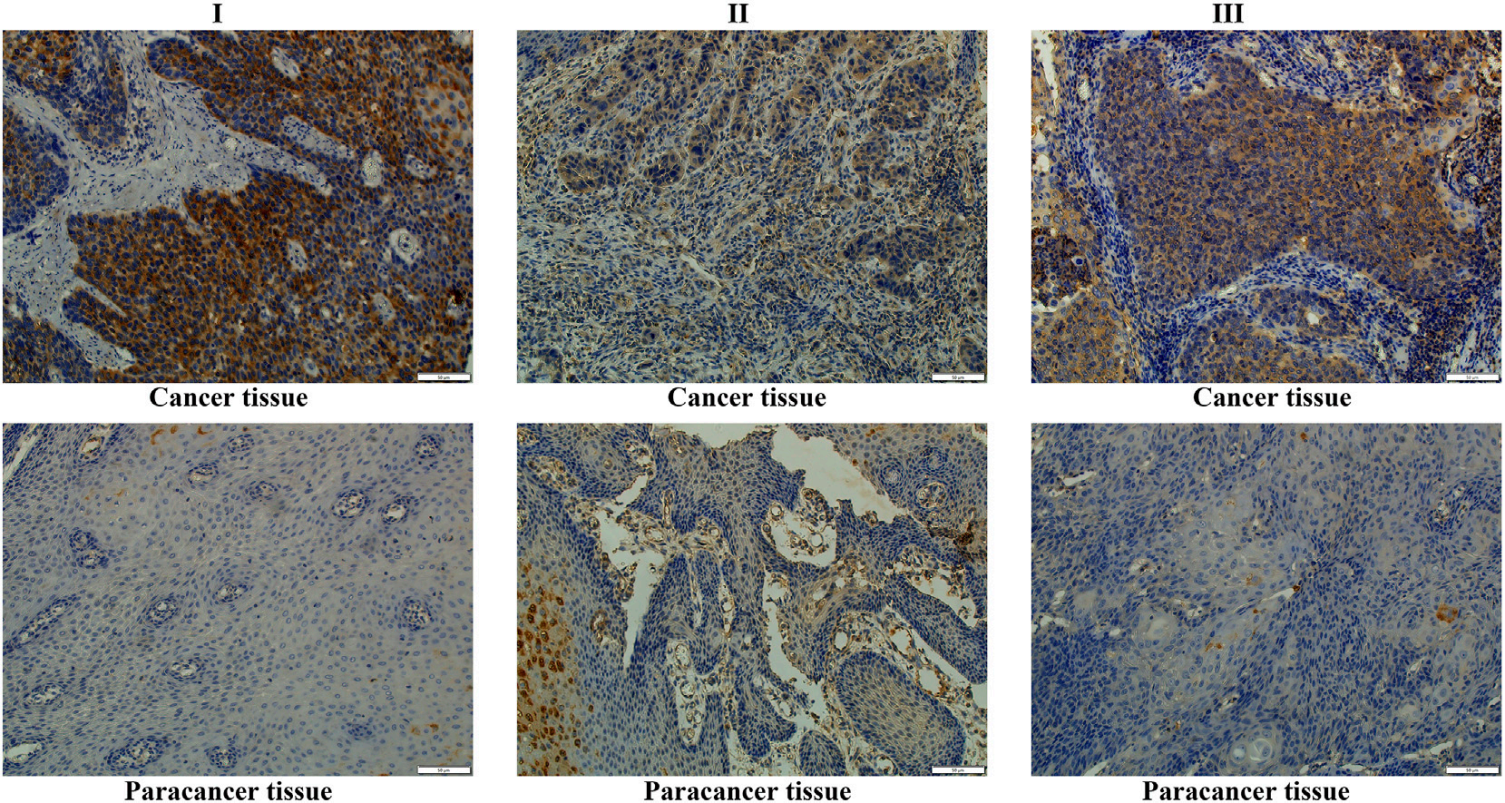
*SPDL1* was overexpressed in clinical ESCA tissues obtained in our hospital. ESCA, esophageal cancer; SPDL1, spindle apparatus coiled-coil protein 1.

In the TCGA and XENA-TCGA-GTEx databases, *SPDL1* expression was increased in both unpaired ESCA patients (Figure 1) and in 8 pairs of ESCA patients (Supplementary Figure S1). In 53 ESCA cases, the SPDL1 protein level was increased in most paired tissues (Figure 2). Among them, *SPDL1* expression level was increased in 44 ESCA tissues and significantly decreased in 9 cancer patients.

### 3.3 Relationship of *SPDL1* Expression With Clinical Features of ESCA

**FIGURE 3:**
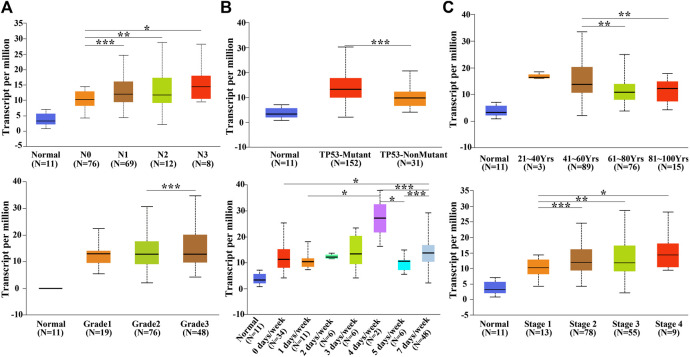
*SPDL1* expression level was related to the clinicopathological features of patients with ESCA in the UALCAN database. **(A)** Lymph node metastasis; **(B)** TP53 mutation; **(C)** age; **(D)** grade; **(E)** drinking history; and **(F)** clinical stage. Normal esophageal tissues; ESCA, esophageal cancer; SPDL1, spindle apparatus coiled-coil protein 1.

The *SPDL1* expression level was evaluated with respect to the clinical features of ESCA patients available on the UALCAN database (Figure 3). *SDPL1* expression level was associated with lymph node metastasis (N0-vs-N1; N0-vs-N2; N0-vs-N3), TP53 mutation (TP53-mutant-vs-TP53-non-mutant), age ((age (41–60yrs)-vs-age (61–80yrs)) and (age (41–60yrs)-vs-age (81–100yrs)), grade (grade2-vs-grade3), drinking history (0 Ddys/week-vs-7 Ddays/week, 1 day/week-vs-4days/week, 4 ay/week-vs-5 days/week, 4 day/week-vs-7 days/week, and 5 day/week-vs-7 days/week), and cancer stage (stage1-vs-stage2, stage1-vs-stage3, and stage1-vs-stage4) of ESCA patients which was significant.

**FIGURE 4:**
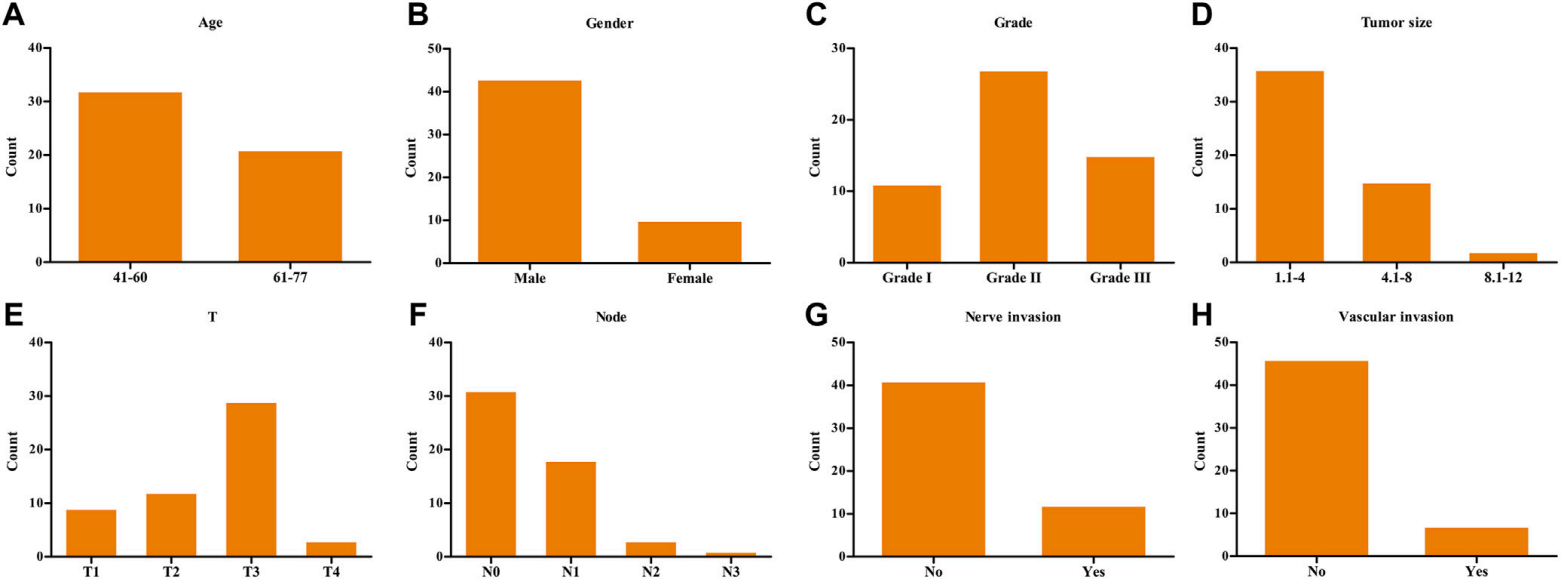
Demographic data of 53 clinical ESCA patients. **(A)** Age; **(B)** gender; **(C)** grade; **(D)** tumor size; **(E)** T stage; **(F)** lymph nodes; **(G)** nerve invasion; and **(H)** vascular invasion. ESCA, esophageal cancer.

The demographic data of 53 ESCA patients are shown in Figure 4. The relationship between *SPDL1* expression level and clinicopathological data (age, gender, grade, T stage, lymph node metastasis, nerve invasion, and vascular invasion) of ESCA patients was investigated. The results were not significant (Table 1). *SPDL1* expression level was significantly correlated with maximum standardized uptake value (SUVmax), SUVmean, and total legion glycolysis (TLG), but was not correlated with metabolic tumor volume, tumor diameter, and tumor invasion depth of PET/CT (Table 2).

**TABLE 1:** Relationship between *SPDL1* expression level and the clinicopathological data of 53 ESCA patients.

Clinical Characteristics	N (53)	%	SPDL1 Expression		P
			Low	High		
Age (year)					0.148
40–60	32		3	29	
61–77	21		6	15	
Gender					0.853
Male	43		8	35	
Female	10		1	9	
Grade					0.439
G1-2	38		5	33	
G3	15		4	11	
T stage					1.000
T1-2	21		4	17	
T3-4	32		5	27	
N stage					1.000
N0	31		5	26	
N1-3	22		4	18	
Nerve invasion					0.686
No	41		6	35	
Yes	12		3	9	
Vascular invasion					
No	46		7	39	0.737
Yes	7		2	5	

Note: ESCA, esophageal cancer; T, tumor size; N, lymph node metastasis; SPDL1, spindle apparatus coiled-coil protein 1.

**TABLE 2:** *SPDL1* overexpression was associated with 18F-FDG PET/CT metabolic parameters of ESCA patients.

PET Metabolic Parameter	SPDL1 Expression		P
	Low (*N* = 9)	High (*N* = 44)	
SUVmax (mean ± SD)	8.7667 ± 1.72860	19.1243 ± 1.23450	0.001
SUVmean (mean ± SD)	5.5144 ± 0.83179	10.6434 ± 0.69459	0.002
TLG (mean ± SD)	18.3378 ± 5.39186	105.468 ± 23.45483	0.001
MTV (mean ± SD)	8.6333 ± 2.42643	7.4536 ± 1.37985	0.718
Diameter	4.4089 ± 0.70621	4.6609 ± 0.32962	0.753
Invasion depth	1.5389 ± 0.18156	1.5364 ± 0.9732	0.991

Note: ESCA, esophageal cancer; PET/CT, positron emission tomography/computed tomography; *SPDL1,* spindle apparatus coiled-coil protein 1.

### 3.4 *SPDL1* Upregulation Was an Adverse Effect on the Poor Prognosis of ESCA Patients

**FIGURE 5:**
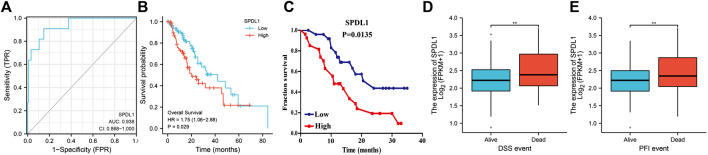
*SPDL1* expression levels in diagnosing ESCA and evaluating the prognosis of patients. **(A)** Diagnostic value; **(B, C)** OS; **(D)** DSS; **(E)** and PFI. OS, overall survival; DSS, disease-specific survival; PFI, progression-free interval; ESCA, esophageal cancer; SPDL1, spindle apparatus coiled-coil protein 1.

*SPDL1* is valuable in the diagnosis and prognosis of ESCA (Figure 5). ROC analysis showed that SPDL1 has the ESCA diagnostic value based on TCGA data (Figure 5A), and the area under the curve of SPDL1 was 0.938. *SPDL1* upregulation represented an adverse effect on the prognosis of ESCA patients based on survival analysis using TCGA database and clinical samples (Figures 5B,C). *SPDL1* expression was significantly abnormal in the disease-specific survival (DSS) and progression-free interval (PFI) events, and was relatively higher in case of deaths (Figures 5D,E).

### 3.5 Biological Functions and Signaling Mechanisms of SPDL1

**FIGURE 6:**
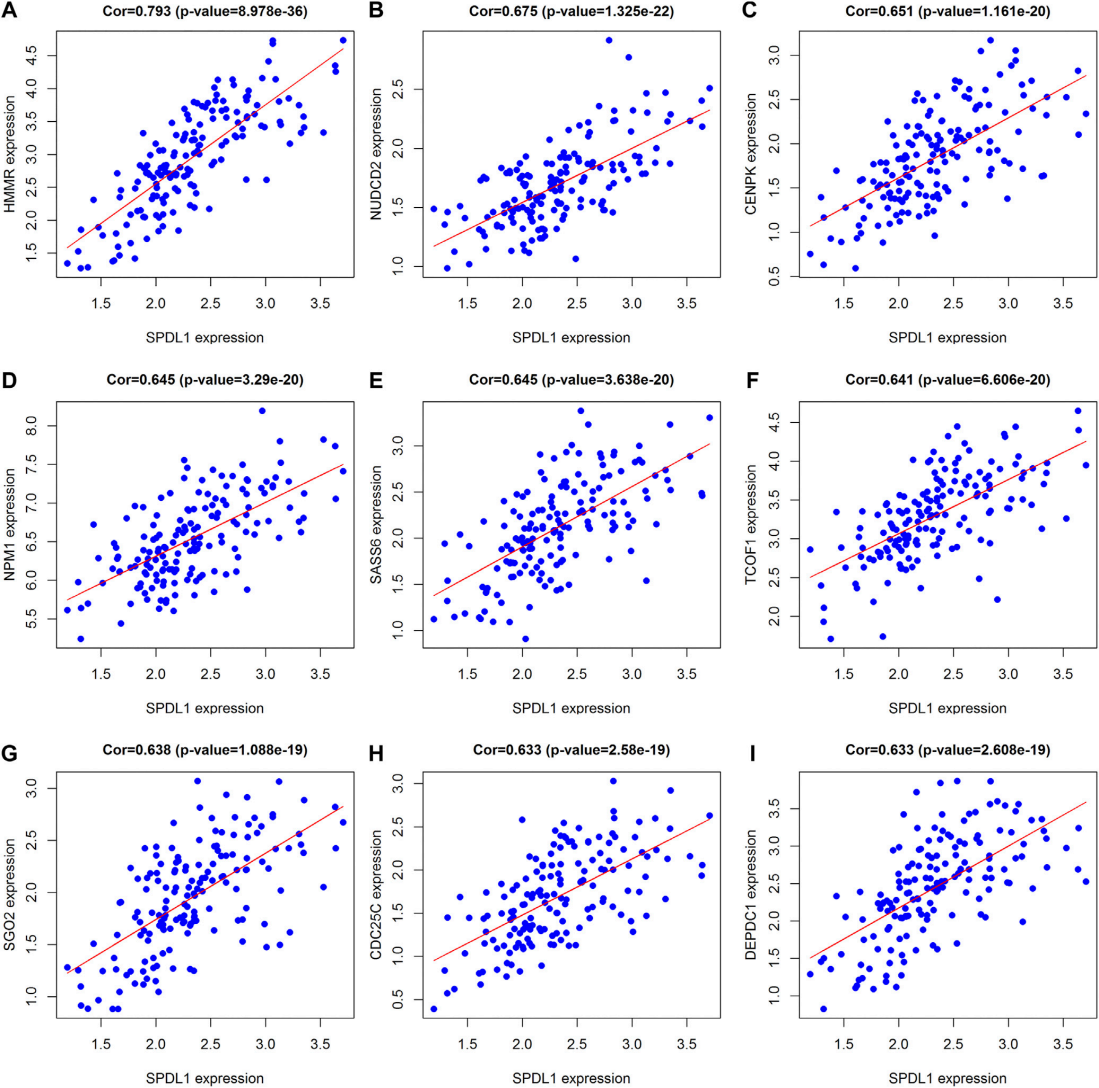
Genes co-expressed with *SPDL1* ranked are shown using correlation coefficient. **(A)** HMMR; **(B)** NUDCD2; **(C)** CENPK; **(D)** NPM1; **(E)** SASS6; **(F)** TCOF1; **(G)** SGO2; **(H)** CDC25C; and **(I)** DEPDC1. ESCA, esophageal cancer tissue; SPDL1, spindle apparatus coiled-coil protein 1.

**FIGURE 7:**
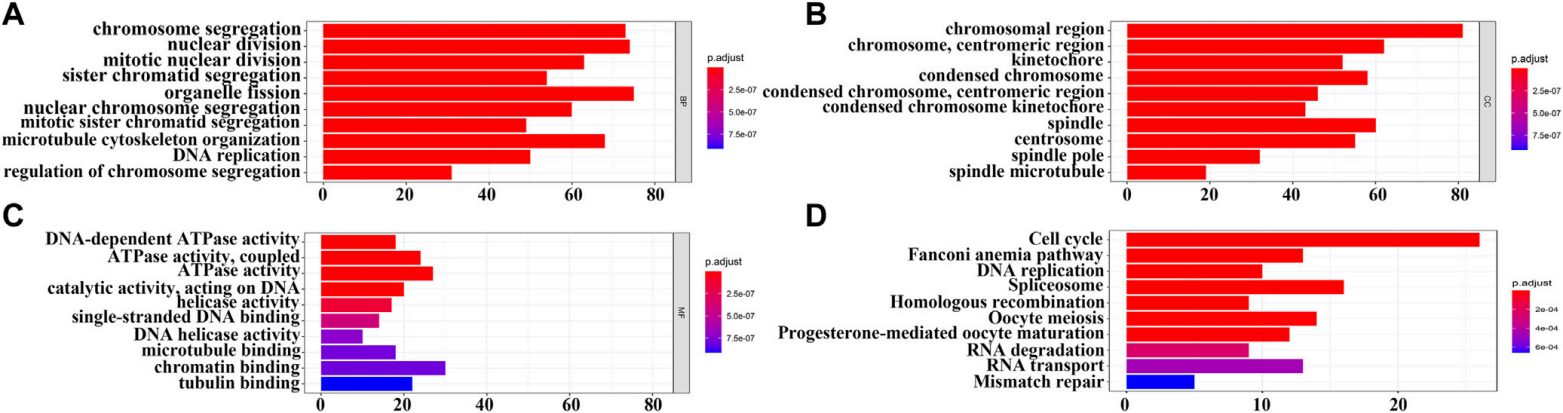
Biological functions and signaling mechanisms of genes co-expressed with *SPDL1* were determined via GO and KEGG analysis. **(A)** BP; **(B)** CC; **(C)** MF; and **(D)** KEGG. BP, biological processes; MF, molecular functions; CC, cellular components; KEGG, Kyoto encyclopedia of genes and genomes; ESCA, esophageal cancer; SPDL1, spindle apparatus coiled-coil protein 1.

**FIGURE 8:**
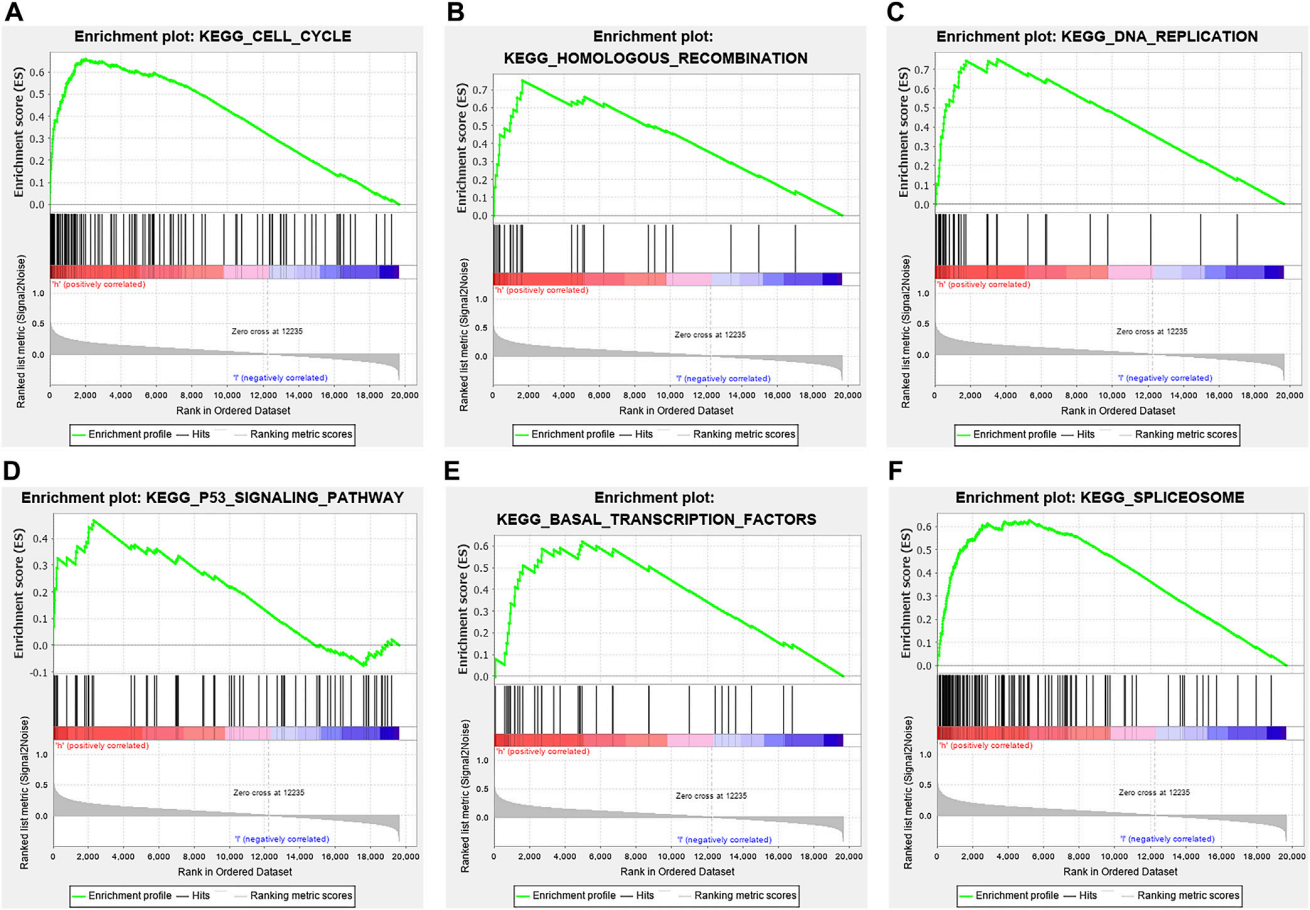
ESCA-related signaling pathways enriched in the high-SPDL1 expression group via GSEA. **(A)** Cell cycle; **(B)** HR; **(C)** DNA replication; **(D)** p53 signaling pathway; **(E)** basal transcription factor; and **(F)** spliceosome. GSEA, gene set enrichment analysis; HR, homologous recombination; ESCA, esophageal cancer; SPDL1, spindle apparatus coiled-coil protein 1.

In total, 393 genes were found to be co-expressed with *SDPL1* (Supplementary Table S1). Figure 6 shows the top 9 co-expressed genes of *SDPL1*. The co-expressed genes mainly participated in chromosome segregation, positive regulation of cell cycle process, DNA replication, cell cycle G2/M phase transition, and meiotic cell cycle based on GO annotation (Figures 7A–C and Supplementary Table S2). KEGG analysis showed that the co-expressed genes mainly participated in DNA replication, homologous recombination (HR), p53 signaling pathway, RNA degradation, and other signaling pathways (Figure 7D and Table 3). GSEA showed that DNA replication, HR, and p53 signaling pathway were highly enriched in the high-SPDL1 expression group (Figure 8 and Table 4).

**TABLE 3:** Genes co-expressed with *SPDL1* involved in signaling pathways based on KEGG analysis.

ID	Description	Count	Adj.p
hsa04110	Cell cycle	26	2.30E-18
hsa03460	Fanconi anemia pathway	13	8.58E-10
hsa03030	DNA replication	10	2.96E-08
hsa03040	Spliceosome	16	5.60E-07
hsa03440	Homologous recombination	9	1.23E-06
hsa04114	Oocyte meiosis	14	2.37E-06
hsa04914	Progesterone-mediated oocyte maturation	12	5.41E-06
hsa03018	RNA degradation	9	0.000242,967
hsa03013	RNA transport	13	0.000461,861
hsa03430	Mismatch repair	5	0.000661,693
hsa05166	Human T-cell leukemia virus 1 infection	13	0.002,755,834
hsa03420	Nucleotide excision repair	5	0.017,361,108
hsa04115	p53 signaling pathway	6	0.021,939,744
hsa04218	Cellular senescence	9	0.027,251,181

Note: ESCA, esophageal cancer; KEGG, Kyoto encyclopedia of genes and genomes; SPDL1, spindle apparatus coiled-coil protein 1.

**TABLE 4:** Mechanisms of upregulated *SPDL1* involved in the occurrence and development of ESCA based on GSEA.

Name	Size	ES	NES	NOM *p*
Oocyte meiosis	112	0.54,050,183	2.340,795	0
Cell cycle	124	0.6,597,191	2.331,743	0
Mismatch repair	23	0.8,347,302	2.2,349,768	0
Homologous recombination	28	0.7,515,696	2.1,316,156	0
Spliceosome	126	0.6,269,084	2.0954,392	0
Basal transcription factors	35	0.62,103,504	2.0752,423	0
Nucleotide excision repair	44	0.6,485,332	2.0345,945	0
Progesterone mediated oocyte maturation	85	0.48,475,447	2.006459	0.00204,499
RNA degradation	57	0.5,889,373	1.9,990,903	0
DNA replication	36	0.75,287,855	1.9,582,809	0.01,010,101
Base excision repair	33	0.6,722,278	1.9,331,809	0.004,301,075
p53 signaling pathway	68	0.46,826,822	1.9,309,136	0
Pyrimidine metabolism	98	0.49,767,083	1.8,963,473	0.004,175,365
Cysteine and methionine metabolism	34	0.519,805	1.7,884,712	0.008,179,959
Purine metabolism	157	0.3,687,451	1.6,533,139	0.014,492,754
One carbon pool by folate	17	0.5,886,974	1.6,212,549	0.04,191,617
Ubiquitin mediated proteolysis	133	0.37,507,394	1.5,899,254	0.02,631,579
RNA polymerase	29	0.52,664,393	1.5,875,642	0.044,176,705

Note: GSEA, gene set enrichment analysis; ES, enrichment score; NES, normalized enrichment score; ESCA, esophageal cancer; *SPDL1*, spindle apparatus coiled-coil protein 1.

### 3.6 Construction of Protein–Protein Interaction Network

A PPI network was constructed using the STRING database (Supplementary Figure S2A). The hub genes in the PPI network were found to be cyclin-dependent kinase 1 (*CDK1*), mitotic checkpoint serine/threonine-protein kinase BUB1 (*BUB1*), G2/mitotic-specific cyclin-B1 (*CCNB1*), BUB1B, cyclin A2 (*CCNA2*), cell division cycle 20 (*CDC20*), mitotic arrest deficient 2 like 1 (*MAD2L1)*, Aurora kinase B (*AURKB*), kinetochore protein NDC80 homolog (*NDC80*), and *PLK1* (Supplementary Figure S2). Correlation analysis showed that *SPDL1* levels and *CDK1, BUB1, CCNB1, BUB1B, CCNA2, CDC20, MAD2L1, AURKB, NDC80*, and *PLK1* levels were significantly correlated (Table 5).

**TABLE 5:** Hub genes in the PPI network.

Gene	Gene Description	Degree	Cor
CDK1	Cyclin-dependent kinases 1	165	0.585
BUB1	BUB1 mitotic checkpoint serine/threonine kinase	148	0.542
CCNB1	Cyclin B1	141	0.606
BUB1B	BUB1 mitotic checkpoint serine/threonine kinase B	135	0.549
CCNA2	Cyclin A2	134	0.576
CDC20	Cell division cycle 20	133	0.501
MAD2L1	Mitotic arrest deficient 2 like 1	132	0.579
AURKB	Aurora kinase B	131	0.491
NDC80	NDC80 kinetochore complex component	125	0.571
PLK1	Polo like kinase 1	125	0.457

Note: PPI, protein–protein interaction.

### 3.7 *SDPL1* Silencing Inhibited ESCA Cell Proliferation and Migration

**FIGURE 9:**
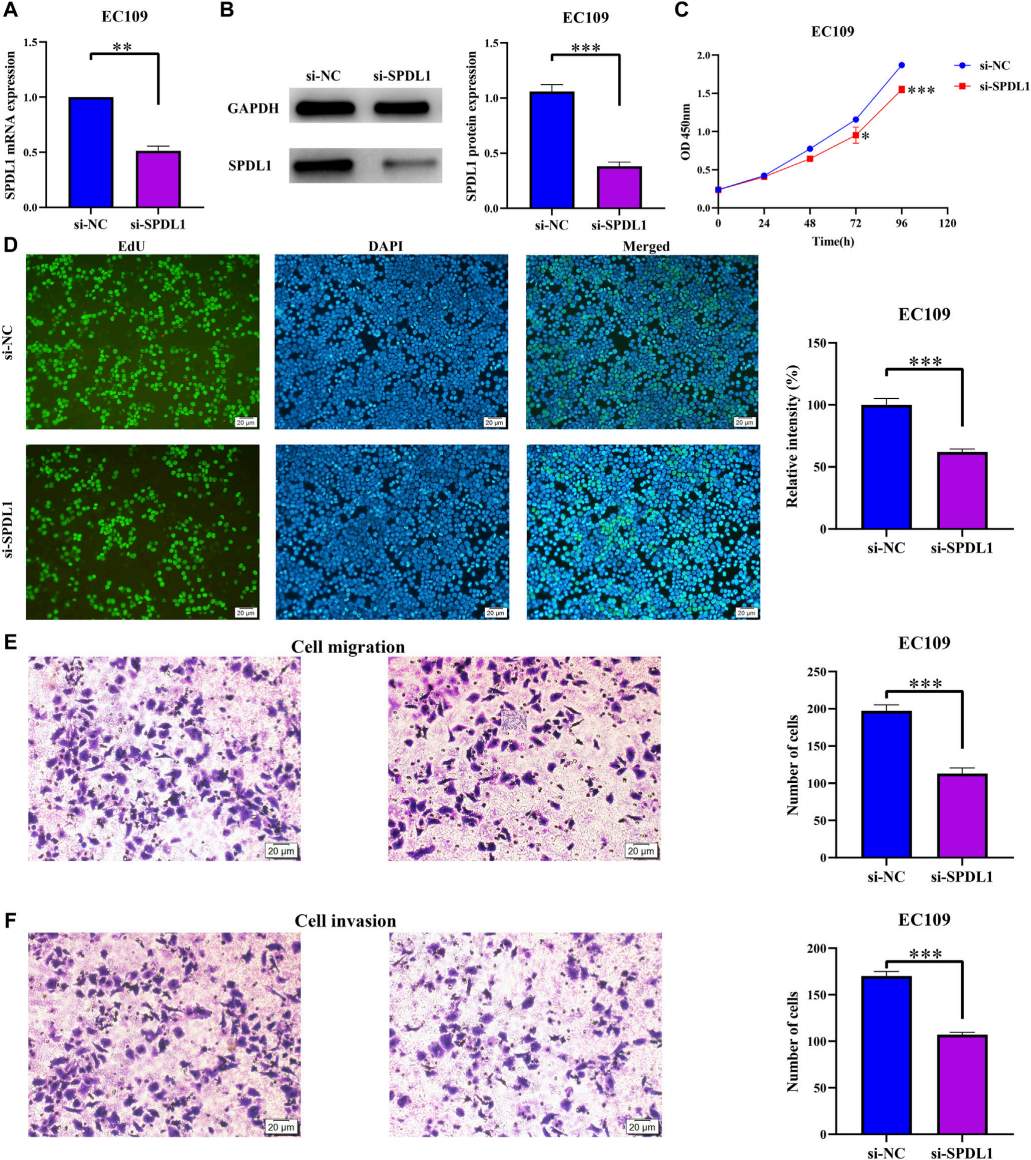
*SPDL1* silencing inhibits ESCA cell growth and migration. **(A,B)** Establishment of ESCA cell model; **(C,D)** cell proliferation determined via Cell Counting Kit-8 and Edu methods and **(E,F)** cell migration and invasion determined using Transwell assays. ESCA, esophageal cancer; SPDL1, spindle apparatus coiled-coil protein 1.

The *SDPL1* mRNA expression level was significantly decreased in the si-SPDL1 group based on qRT-PCR analysis (Figure 9A). The sequence of *SPDL1* which showed the best interference was selected to verify the protein expression of SPDL1 in si-SPDL1 and si-NC groups via WB. SPDL1 protein level was significantly decreased in the si-SPDL1 group (Figure 9B). CCK-8 and Edu analyses showed that cell proliferation was significantly decreased after *SPDL1* silencing. Cells in the si-SPDL1 group showed inhibited cell proliferation (Figures 9C,D). Transwell analyses showed that cells in the si-SPDL1 group showed significantly decreased cell migration and invasion abilities (Figures 9E,F).

## 4 Discussion

The five-year survival time of patients with ESCA remains low due to the lack of target molecules for early diagnosis, drug treatment, and evaluation of prognosis of ESCA. Consequently, new biomarkers are sought after to improve the overall survival of ESCA patients. *SPDL1* has been found to play a role in cancer progression; however, its role in ESCA remains unknown. Under physiological conditions, mitosis plays an important role in maintaining normal cell growth and development. Abnormal mitosis is closely associated with cell proliferation and apoptosis (Kalimutho et al., 2018; Matsunuma et al., 2018; Liu et al., 2019). For example, in dihydropyrimidinase-like 3 (DPYSL3)-positive breast cancer CLOW cells, cell proliferation decreases and the expression of marker genes in epithelial-mesenchymal transformation increases in the *DPYSL3* knockdown group. The low proliferation of DPYSL3-negative CLOW cells is associated with the accumulation of multinucleated cells, suggesting that it is associated with mitotic defects and increased vimentin levels and vimentin phosphorylation (Matsunuma et al., 2018). SPDL1 plays an important role in mitotic spindle formation and chromosome segregation (Barisic et al., 2010; Kodama et al., 2019). Tian et al. reported that abnormal spindle-like microcephaly-associated protein, *BUB1B*, and *SPDL1* are highly expressed in pancreatic ductal adenocarcinoma and are associated with poor OS and disease-free survival (Tian and Wang, 2020). Our results revealed that *SPDL1* expression was increased in ESCA tissues based on TCGA database and clinical samples, and was associated with lymph node metastasis, TP53 mutation, age, grade, drinking history, and cancer stage of ESCA patients based on bioinformatics analysis. SPDL1 was found to have an ESCA diagnostic value, and *SPDL1* upregulation had an adverse effect on the prognosis of ESCA patients. In 53 ESCA patients, *SPDL1* expression was significantly correlated with SUVmax, SUVmean, and TLG of PET/CT, and was not related to the age, gender, grade, T stage, lymph node metastasis, nerve invasion, and vascular invasion. These results indicate that SPDL1 may serve as a diagnostic and prognostic marker of ESCA.

SPDL1 is a target molecule downstream of MRTFB. MRTFB inhibits the invasion and migration of CRC cells. However, disruption of *SPDL1* expression in CRC cells significantly increases invasion and migration. Disruption of *MRTFB* expression suppresses *SPDL1* expression in the intestinal tract of mice. *SPDL1* expression has been found to be significantly correlated to the survival rate of mice. Disruption of *SPDL1* expression promotes the development of xenografts in nude mice (Kodama et al., 2019). In our cell model, interference with *SPDL1* expression inhibited ESCA cell growth and migration of cells transfected with *SPDL1* siRNA. In addition, cell cycle, HR, DNA replication, and p53 signaling pathway, which were associated with *SPDL1* expression, represent the most common mechanisms of cancer progression (Zou et al., 2016; Fu et al., 2018; Lv et al., 2018; Mahajan et al., 2019). For example, Fu et al. reported that ARC15 and ARC17 induce apoptosis in colon cancer cells by increasing PUMA expression and activating mitochondria, which is related to cell cycle arrest induced by increased p21 expression, inhibition of proteasome activity and MDM2 expression, and p53 activation and accumulation (Fu et al., 2018). Cell proliferation has been found to increase with abnormal checkpoint expression when cancer cells show HR deficiency. Tumor suppressor proteins BRCA2 and p53 checkpoint regulators may counteract abnormal cell proliferation. RAD52 attenuates the inhibition of HR in BRCA2-deficient cells via p53 (Mahajan et al., 2019). In our study, *SPDL1* was significantly associated with mitotic nuclear division, DNA replication, cell cycle, HR, and p53 signaling pathway based on GO and KEGG analyses, and *SPDL1* silencing inhibited ESCA cell growth and migration. These results indicated that SPDL1 may participate in the progression of ESCA via regulation of the cell cycle, HR, DNA replication, and p53 signaling pathway. The mechanisms associated with the role of SPDL1 in ESCA progression should be investigated in future studies.

At present, many studies have shown that the hub genes *CDC20, CDK1, BUB1, CCNB1*, and *BUB1B* identified in our PPI network are involved in cancer progression (Zhang et al., 2018; Piao et al., 2019; Li et al., 2020; Zhang et al., 2020; Gao et al., 2021). *CCNB1* expression is relatively higher in pancreatic cancer tissues. *CCNB1* expression in shCCNB1-transfected cells is relatively lower, the ratio of proliferating and S phase cells in shCCNB1-transfected cells is decreased, and the ratio of apoptosis, senescence, and G0/G1 phase cells is increased. *CCNB1* silencing inhibits cell proliferation and promotes cell senescence by activating the p53 signaling pathway (Zhang et al., 2018). Overexpression of *CDC20* is associated with poor prognosis in patients with osteosarcoma. Downregulation of *CDC20* inhibits the proliferation of osteosarcoma cells and induces apoptosis and cell cycle arrest. *CDC20* overexpression promotes cell growth and inhibits cell apoptosis (Gao et al., 2021). Therefore, SPDL1 may be of high value in cancer progression.

In conclusion, *SPDL1* was overexpressed in ESCA tissues. Increased *SPDL1* expression was associated with diagnosis and poor prognosis of ESCA patients. *SPDL1* overexpression was associated with 18F-FDG PET/CT metabolic parameters, where ESCA patients that showed high PET/CT SUVmax, SUVmean, and TLG had a poor prognosis. However, we found that the two were not significant when the relationship between PET/CT metabolic parameters (SUVmax, SUVmean, and TLG) and the prognosis of ESCA patients were analyzed. *SPDL1* was upregulated in ESCA, and increased *SPDL1* expression was associated with poor prognosis, and cancer progression. SPDL1 may regulate ESCA progression via the cell cycle, HR, DNA replication, and p53 signaling pathway.

## Data Availability

The data analyzed in this study could be obtained in TCGA (https://portal.gdc.cancer.gov/projects/) and GTEx (https://gtexportal.org/home/datasets) databases, and relevant research materials could be obtained from the corresponding author.

## Abbreviations

ESCA, esophageal cancer; TIMER, Tumor Immune Estimation Resource; TCGA, The Cancer Genome Atlas; GO, Gene Ontology; KEGG, Kyoto Encyclopedia of Genes and Genomes; GSEA, Gene Set Enrichment Analysis; PPI, protein–protein interaction; OS, overall survival; BP, biological processes; MF, molecular functions; CC, cellular components; NES, Nominal enrichment scores; SPDL1, spindle apparatus coiled-coil protein 1.
