# Correction: SPDL1 overexpression is associated with the 18F-FDG PET/CT metabolic parameters, prognosis, and progression of esophageal cancer

**DOI:** 10.3389/fgene.2026.1848575

**Published:** 2026-05-20

**Authors:** Hua-Song Liu, Qiang Guo, Heng Yang, Min Zeng, Li-Qiang Xu, Qun-Xian Zhang, Hua Liu, Jia-Long Guo, Jun Zhang

**Affiliations:** Department of Cardiothoracic Surgery, Taihe Hospital, Hubei University of Medicine, Shiyan, China

**Keywords:** spindle apparatus coiled-coil protein 1, esophageal cancer, prognosis, biomarker, positron emission tomography/computed tomography

There was a mistake in Figure 9 as published. The representative images in Figures 9E,F of the control group cells were duplicated. Upon re-examination of the raw data, we found no issues with the control cells in the migration assay. However, the control cells in the invasion assay required re-counting. In this erratum, we re-counted the control cells in the invasion assay and observed a statistically significant difference between the experimental and control groups. This indicated that suppressing SPDL1 expression inhibits cell invasion, which was consistent with our previous results. The corrected Figure 9 and its caption appears below.

**FIGURE 9:**
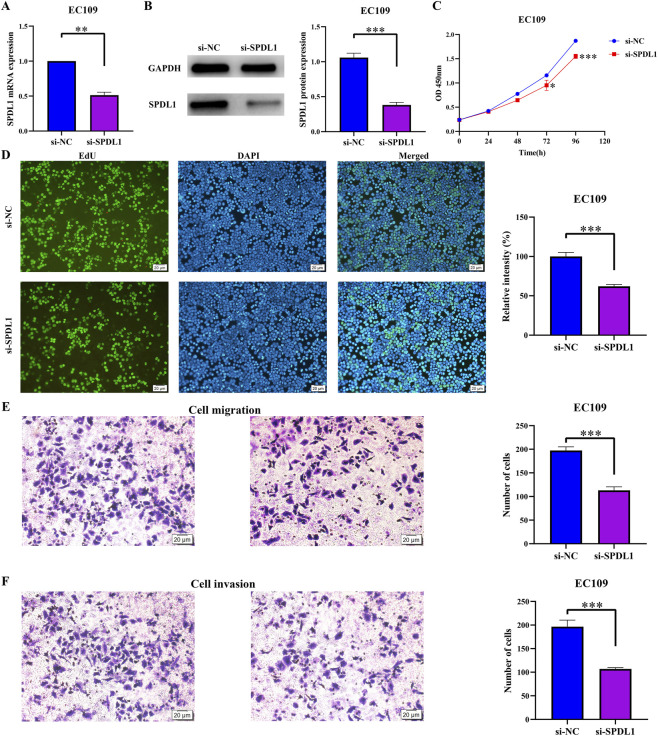
SPDL1 silencing inhibits ESCA cell growth and migration. **(A,B)** Establishment of ESCA cell model; **(C,D)** cell proliferation determined via Cell Counting Kit-8 and Edu methods and **(E,F)** cell migration and invasion determined using Transwell assays. ESCA, esophageal cancer; SPDL1, spindle apparatus coiled-coil protein 1.

The original article has been updated.

