# Supplementary material for: SPDL1 Overexpression Is Associated With the 18F-FDG PET/CT Metabolic Parameters, Prognosis, and Progression of Esophageal Cancer
Source: Front Genet. 2022 May 18;13:798020. doi: 10.3389/fgene.2022.798020 (PMC9157543; doi:10.3389/fgene.2022.798020)
Supplement: Supplementary file 1 [file DataSheet1.doc]

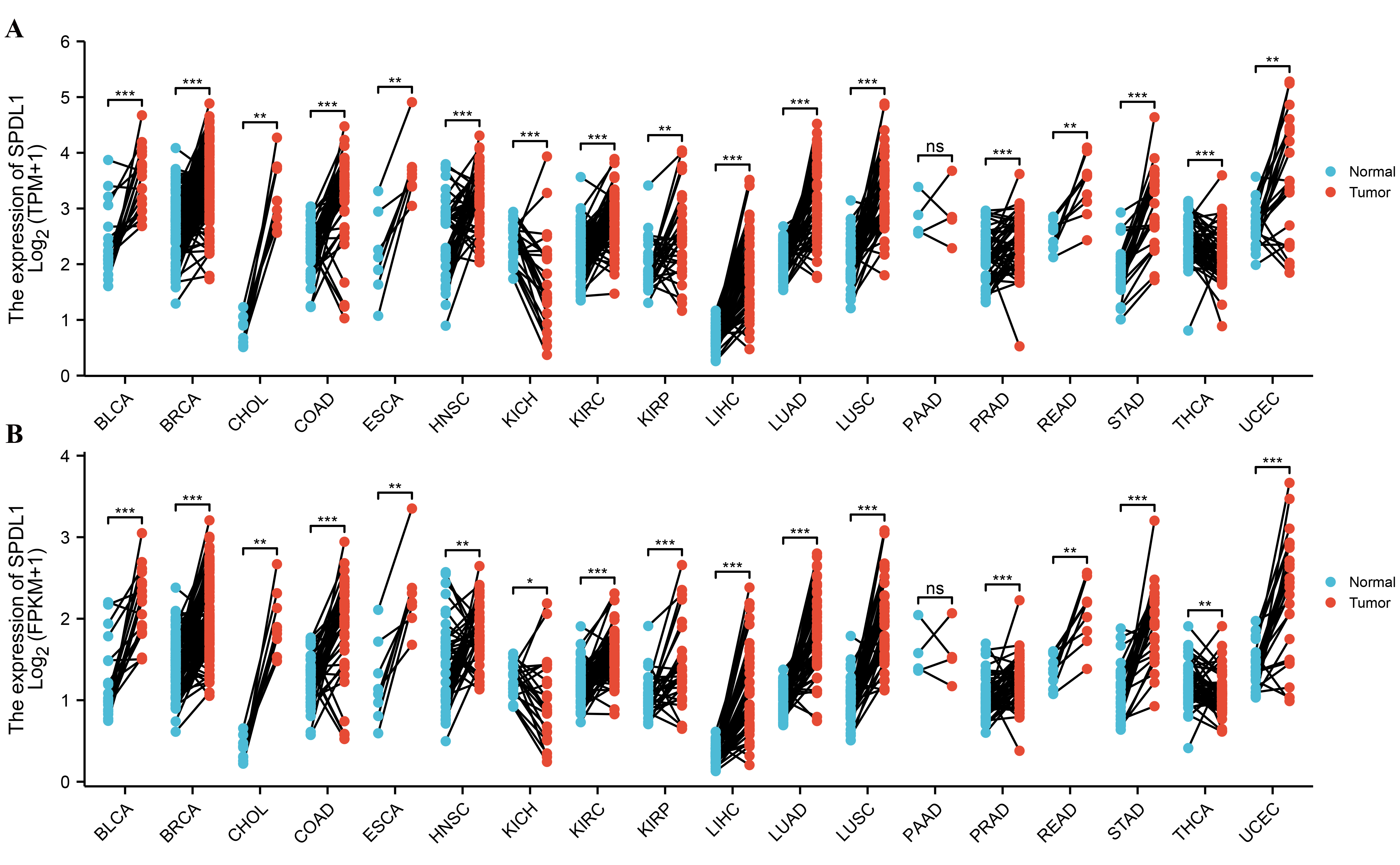


Figure S1. The pan-cancer data of TCGA database were found that SPDL1 was abnormally expressed in a variety of matched cancer tissues.

Note: TCGA, The Cancer Genome Atlas; *, P < 0.05; **, P < 0.01; ***, P < 0.001.


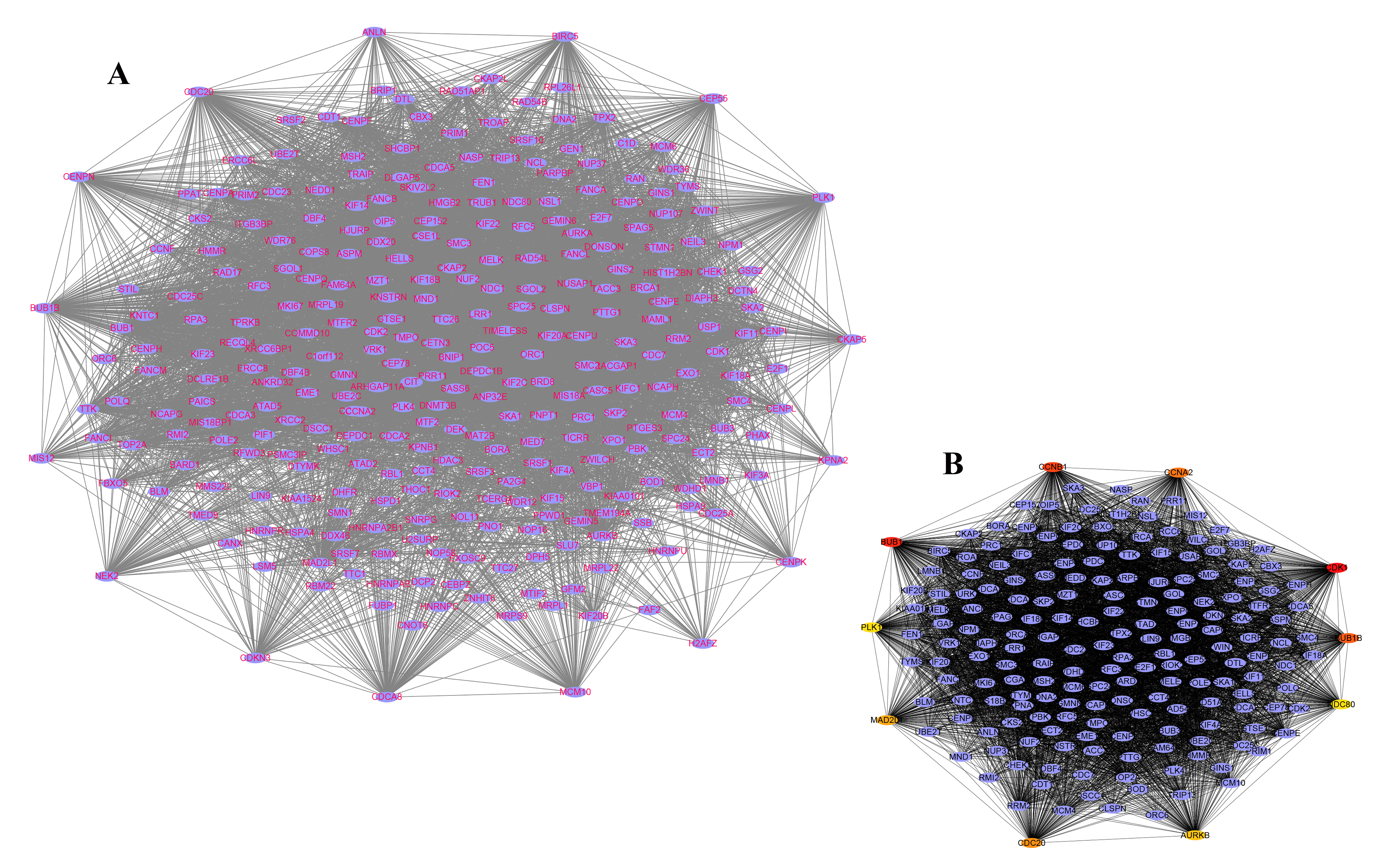


Figure S2. PPI network of SPDL1 co-expression genes. (A) PPI network; (B) Hub genes in PPI network.

Note: PPI, Protein-protein interaction.

Table S1. SPDL1 co-expressed genes in TCGA.

| CorGene | cor | pvalue | CorGene | cor | pvalue | CorGene | cor | pvalue |
| --- | --- | --- | --- | --- | --- | --- | --- | --- |
| HMMR | 0.793 | 8.98E-36 | MCM10 | 0.494 | 3.34E-11 | BRCA1 | 0.442 | 4.90E-09 |
| NUDCD2 | 0.675 | 1.32E-22 | CHEK1 | 0.494 | 3.17E-11 | MIS12 | 0.441 | 5.21E-09 |
| CENPK | 0.651 | 1.16E-20 | TRAIP | 0.493 | 3.54E-11 | DBF4 | 0.441 | 5.13E-09 |
| NPM1 | 0.645 | 3.29E-20 | VRK1 | 0.493 | 3.56E-11 | PPWD1 | 0.44 | 5.58E-09 |
| SASS6 | 0.645 | 3.64E-20 | PRIM2 | 0.493 | 3.62E-11 | NOP16 | 0.44 | 5.84E-09 |
| TCOF1 | 0.641 | 6.61E-20 | RAD17 | 0.492 | 4.04E-11 | C5orf15 | 0.44 | 5.75E-09 |
| SGO2 | 0.638 | 1.09E-19 | AURKB | 0.491 | 4.42E-11 | ORC1 | 0.44 | 5.68E-09 |
| CDC25C | 0.633 | 2.58E-19 | NCL | 0.491 | 4.26E-11 | FAM161A | 0.439 | 6.26E-09 |
| DEPDC1 | 0.633 | 2.61E-19 | PRC1 | 0.491 | 4.56E-11 | DCTN4 | 0.439 | 6.42E-09 |
| KIF18A | 0.632 | 3.22E-19 | NEMP1 | 0.491 | 4.53E-11 | CEP78 | 0.438 | 7.02E-09 |
| KIF4A | 0.624 | 1.27E-18 | BRD8 | 0.49 | 4.64E-11 | CEP55 | 0.438 | 6.92E-09 |
| HJURP | 0.618 | 3.19E-18 | TYMS | 0.49 | 4.64E-11 | WDR76 | 0.437 | 7.46E-09 |
| FBXO5 | 0.615 | 5.25E-18 | ECT2 | 0.49 | 4.72E-11 | EXTL2 | 0.437 | 7.68E-09 |
| KIF20A | 0.613 | 7.16E-18 | KIF22 | 0.489 | 5.47E-11 | KIF3A | 0.436 | 7.96E-09 |
| SLF1 | 0.608 | 1.48E-17 | CCT4 | 0.488 | 5.74E-11 | HNRNPC | 0.436 | 8.39E-09 |
| CCNB1 | 0.606 | 2.07E-17 | CIT | 0.488 | 5.72E-11 | TRIP13 | 0.436 | 8.18E-09 |
| NCAPG | 0.605 | 2.25E-17 | MIS18A | 0.487 | 6.33E-11 | GPN3 | 0.436 | 8.32E-09 |
| DLGAP5 | 0.605 | 2.34E-17 | KIF20B | 0.487 | 6.56E-11 | HNRNPU | 0.435 | 9.37E-09 |
| CENPA | 0.6 | 5.39E-17 | HELLS | 0.487 | 6.66E-11 | E2F1 | 0.435 | 9.16E-09 |
| PLK4 | 0.596 | 8.67E-17 | XRCC2 | 0.486 | 7.66E-11 | MCM4 | 0.434 | 9.60E-09 |
| ERCC6L | 0.593 | 1.38E-16 | CBX3 | 0.486 | 7.51E-11 | HSPA4 | 0.434 | 9.56E-09 |
| KIF18B | 0.592 | 1.64E-16 | CENPQ | 0.485 | 8.32E-11 | NUP107 | 0.434 | 9.89E-09 |
| ARHGAP11A | 0.587 | 3.56E-16 | CENPF | 0.484 | 8.77E-11 | ZNF346 | 0.433 | 1.03E-08 |
| POC5 | 0.586 | 3.74E-16 | PSMC3IP | 0.484 | 9.39E-11 | SLU7 | 0.433 | 1.03E-08 |
| CDK1 | 0.585 | 4.39E-16 | MCM6 | 0.484 | 8.87E-11 | KPNB1 | 0.432 | 1.21E-08 |
| SKA3 | 0.585 | 4.37E-16 | NIF3L1 | 0.483 | 9.95E-11 | DPH5 | 0.432 | 1.17E-08 |
| GMNN | 0.581 | 8.38E-16 | RFC5 | 0.483 | 1.01E-10 | BORA | 0.431 | 1.26E-08 |
| ORC6 | 0.58 | 8.62E-16 | KNTC1 | 0.483 | 9.49E-11 | TMPO | 0.431 | 1.26E-08 |
| LMNB1 | 0.579 | 1.13E-15 | ZWINT | 0.483 | 9.94E-11 | ZNF367 | 0.43 | 1.44E-08 |
| FAF2 | 0.579 | 1.05E-15 | CDCA2 | 0.481 | 1.21E-10 | ZGRF1 | 0.43 | 1.35E-08 |
| MAD2L1 | 0.579 | 1.03E-15 | G3BP1 | 0.481 | 1.27E-10 | SMN1 | 0.43 | 1.42E-08 |
| CENPE | 0.579 | 1.08E-15 | SLC38A9 | 0.481 | 1.23E-10 | MRPL1 | 0.429 | 1.49E-08 |
| CCNA2 | 0.576 | 1.67E-15 | RACGAP1 | 0.48 | 1.32E-10 | HSPD1 | 0.429 | 1.52E-08 |
| DEPDC1B | 0.575 | 1.82E-15 | SHCBP1 | 0.48 | 1.34E-10 | MMS22L | 0.429 | 1.49E-08 |
| SPC25 | 0.575 | 1.87E-15 | MRPL19 | 0.479 | 1.51E-10 | CFAP36 | 0.428 | 1.71E-08 |
| SGO1 | 0.575 | 1.74E-15 | SRSF10 | 0.478 | 1.67E-10 | TACC3 | 0.428 | 1.70E-08 |
| KIF2C | 0.572 | 2.83E-15 | DEK | 0.478 | 1.61E-10 | CENPU | 0.428 | 1.60E-08 |
| DBF4B | 0.572 | 2.75E-15 | MZT1 | 0.478 | 1.72E-10 | GPR19 | 0.428 | 1.58E-08 |
| TTK | 0.571 | 3.30E-15 | UBE2T | 0.477 | 1.85E-10 | ZFP62 | 0.428 | 1.64E-08 |
| NDC80 | 0.571 | 3.27E-15 | SFXN1 | 0.475 | 2.25E-10 | GAS2L3 | 0.427 | 1.82E-08 |
| UBTD2 | 0.568 | 4.76E-15 | WDHD1 | 0.475 | 2.16E-10 | FIGNL1 | 0.427 | 1.84E-08 |
| XPO1 | 0.568 | 4.97E-15 | TEX30 | 0.474 | 2.46E-10 | HMGXB3 | 0.427 | 1.82E-08 |
| FAM72B | 0.568 | 4.57E-15 | FUBP1 | 0.474 | 2.45E-10 | PCLAF | 0.427 | 1.83E-08 |
| UBE2C | 0.567 | 5.62E-15 | GEMIN6 | 0.474 | 2.32E-10 | SERBP1 | 0.426 | 1.93E-08 |
| BIRC5 | 0.566 | 6.06E-15 | TOP2A | 0.474 | 2.35E-10 | GFM2 | 0.426 | 1.94E-08 |
| BOD1 | 0.564 | 7.95E-15 | PAICS | 0.474 | 2.40E-10 | CHORDC1 | 0.424 | 2.22E-08 |
| TROAP | 0.561 | 1.15E-14 | CDC25A | 0.473 | 2.65E-10 | NUP37 | 0.424 | 2.31E-08 |
| PTTG1 | 0.561 | 1.22E-14 | CKAP2 | 0.473 | 2.73E-10 | CHML | 0.424 | 2.33E-08 |
| RPL26L1 | 0.56 | 1.44E-14 | FANCM | 0.472 | 2.87E-10 | ARMC1 | 0.424 | 2.23E-08 |
| NEK2 | 0.559 | 1.66E-14 | RPAP3 | 0.471 | 3.18E-10 | AUNIP | 0.423 | 2.61E-08 |
| KIFC1 | 0.557 | 2.14E-14 | DNA2 | 0.471 | 3.42E-10 | NSL1 | 0.423 | 2.59E-08 |
| CDC23 | 0.557 | 2.08E-14 | TRIM59 | 0.471 | 3.29E-10 | WDCP | 0.423 | 2.57E-08 |
| PSRC1 | 0.556 | 2.35E-14 | ALG10 | 0.471 | 3.29E-10 | LIN9 | 0.423 | 2.57E-08 |
| TPX2 | 0.556 | 2.35E-14 | RFC3 | 0.47 | 3.63E-10 | DDX41 | 0.423 | 2.45E-08 |
| CLSPN | 0.555 | 2.67E-14 | ZCCHC9 | 0.47 | 3.52E-10 | MTREX | 0.423 | 2.52E-08 |
| RARS | 0.552 | 3.68E-14 | LRR1 | 0.47 | 3.60E-10 | SRFBP1 | 0.422 | 2.68E-08 |
| CDCA3 | 0.552 | 3.70E-14 | CEP152 | 0.469 | 3.91E-10 | FANCL | 0.422 | 2.82E-08 |
| MSH2 | 0.549 | 5.54E-14 | BRIP1 | 0.469 | 3.96E-10 | SKP2 | 0.421 | 2.98E-08 |
| NCAPH | 0.549 | 5.96E-14 | BARD1 | 0.468 | 4.28E-10 | TTC27 | 0.421 | 2.94E-08 |
| BUB1B | 0.549 | 5.63E-14 | COPS8 | 0.468 | 4.33E-10 | E2F7 | 0.421 | 2.97E-08 |
| CDC7 | 0.548 | 6.64E-14 | BUB3 | 0.468 | 4.59E-10 | MTF2 | 0.421 | 2.93E-08 |
| KIF23 | 0.548 | 6.77E-14 | SKA2 | 0.468 | 4.37E-10 | DENR | 0.421 | 3.00E-08 |
| ANLN | 0.544 | 1.01E-13 | SMC2 | 0.467 | 4.80E-10 | TIMELESS | 0.421 | 3.01E-08 |
| DHFR | 0.544 | 1.07E-13 | CKAP2L | 0.467 | 4.62E-10 | PTGES3 | 0.421 | 3.03E-08 |
| BUB1 | 0.542 | 1.30E-13 | MIS18BP1 | 0.467 | 5.05E-10 | C2orf49 | 0.42 | 3.30E-08 |
| ZBED8 | 0.537 | 2.59E-13 | DCLRE1B | 0.466 | 5.20E-10 | CRIPT | 0.42 | 3.17E-08 |
| MRPL22 | 0.537 | 2.58E-13 | FEN1 | 0.465 | 5.96E-10 | MORN2 | 0.42 | 3.26E-08 |
| KIF15 | 0.536 | 2.71E-13 | NOP58 | 0.465 | 5.99E-10 | OTUD6B | 0.42 | 3.31E-08 |
| RAD51AP1 | 0.535 | 3.27E-13 | ERCC8 | 0.464 | 6.29E-10 | CEP128 | 0.42 | 3.17E-08 |
| EME1 | 0.534 | 3.38E-13 | SNRPG | 0.464 | 6.14E-10 | RAPGEF6 | 0.419 | 3.47E-08 |
| CDCA8 | 0.534 | 3.45E-13 | POLQ | 0.464 | 6.30E-10 | ARL6IP6 | 0.419 | 3.58E-08 |
| CNOT6 | 0.532 | 4.69E-13 | FANCA | 0.463 | 7.27E-10 | STIL | 0.419 | 3.43E-08 |
| ASPM | 0.531 | 4.97E-13 | CDK2 | 0.463 | 6.87E-10 | TRMT13 | 0.418 | 3.93E-08 |
| RBM27 | 0.53 | 5.91E-13 | CDT1 | 0.463 | 7.23E-10 | DDX20 | 0.418 | 3.86E-08 |
| ATAD2 | 0.529 | 6.33E-13 | RPA3 | 0.462 | 7.57E-10 | DHX57 | 0.418 | 3.68E-08 |
| BLM | 0.529 | 6.80E-13 | MTIF2 | 0.462 | 7.64E-10 | PHF6 | 0.418 | 3.69E-08 |
| CCDC18 | 0.528 | 7.49E-13 | LRRC40 | 0.462 | 7.54E-10 | SAAL1 | 0.418 | 3.78E-08 |
| KIF14 | 0.528 | 7.59E-13 | CCDC43 | 0.462 | 7.56E-10 | TTC26 | 0.417 | 4.08E-08 |
| AURKA | 0.526 | 9.14E-13 | TBC1D31 | 0.46 | 9.74E-10 | ANP32E | 0.416 | 4.37E-08 |
| CETN3 | 0.525 | 9.71E-13 | DDX46 | 0.46 | 9.66E-10 | DTL | 0.416 | 4.35E-08 |
| EXO1 | 0.525 | 1.01E-12 | OIP5 | 0.46 | 9.00E-10 | SAMD15 | 0.416 | 4.42E-08 |
| CENPL | 0.525 | 1.01E-12 | ATAD5 | 0.459 | 1.01E-09 | LYRM7 | 0.415 | 4.68E-08 |
| PRIM1 | 0.524 | 1.14E-12 | ZNHIT6 | 0.459 | 1.03E-09 | ZFP1 | 0.414 | 5.19E-08 |
| NUF2 | 0.522 | 1.51E-12 | PPAT | 0.458 | 1.12E-09 | H2AFZ | 0.414 | 5.16E-08 |
| MELK | 0.521 | 1.59E-12 | PBK | 0.458 | 1.11E-09 | WDR12 | 0.413 | 5.57E-08 |
| MRPS9 | 0.517 | 2.46E-12 | MAT2B | 0.458 | 1.16E-09 | KHDC1 | 0.413 | 5.55E-08 |
| SPAG5 | 0.516 | 2.95E-12 | NUDCD1 | 0.457 | 1.24E-09 | GEN1 | 0.413 | 5.49E-08 |
| BNIP1 | 0.516 | 2.88E-12 | PLK1 | 0.457 | 1.29E-09 | SMC3 | 0.413 | 5.74E-08 |
| NEIL3 | 0.516 | 3.04E-12 | POLE2 | 0.457 | 1.28E-09 | RMI2 | 0.413 | 5.67E-08 |
| KIF11 | 0.515 | 3.29E-12 | FAM72A | 0.456 | 1.42E-09 | PHAX | 0.412 | 6.19E-08 |
| ITGB3BP | 0.515 | 3.17E-12 | FAM111B | 0.456 | 1.34E-09 | SSX2IP | 0.412 | 6.02E-08 |
| PRR11 | 0.515 | 3.36E-12 | G2E3 | 0.456 | 1.39E-09 | MED7 | 0.412 | 6.28E-08 |
| CENPI | 0.514 | 3.67E-12 | PARPBP | 0.455 | 1.45E-09 | AGGF1 | 0.411 | 6.86E-08 |
| KNSTRN | 0.513 | 4.08E-12 | SRSF3 | 0.455 | 1.45E-09 | POC1A | 0.411 | 6.90E-08 |
| HMGB2 | 0.513 | 4.24E-12 | SPC24 | 0.455 | 1.54E-09 | GPR180 | 0.411 | 6.53E-08 |
| PIF1 | 0.512 | 4.68E-12 | RRM2 | 0.455 | 1.53E-09 | SRSF2 | 0.411 | 6.88E-08 |
| TICRR | 0.512 | 4.37E-12 | GINS1 | 0.454 | 1.68E-09 | ZCCHC10 | 0.411 | 6.76E-08 |
| SRSF1 | 0.512 | 4.51E-12 | SUV39H2 | 0.454 | 1.61E-09 | VBP1 | 0.411 | 6.73E-08 |
| CENPH | 0.51 | 5.77E-12 | SKA1 | 0.454 | 1.58E-09 | ATG4C | 0.41 | 7.21E-08 |
| CENPN | 0.51 | 5.48E-12 | LSM5 | 0.454 | 1.71E-09 | TRUB1 | 0.409 | 7.82E-08 |
| CDKN3 | 0.509 | 6.26E-12 | SRSF7 | 0.453 | 1.84E-09 | RECQL4 | 0.409 | 8.04E-08 |
| CANX | 0.508 | 7.40E-12 | HASPIN | 0.453 | 1.86E-09 | STMN1 | 0.408 | 8.88E-08 |
| USP1 | 0.507 | 7.97E-12 | C1orf112 | 0.453 | 1.75E-09 | HDAC3 | 0.408 | 8.31E-08 |
| CDCA5 | 0.506 | 9.25E-12 | RBMX | 0.452 | 2.02E-09 | PA2G4 | 0.408 | 8.33E-08 |
| RAD54L | 0.506 | 8.32E-12 | NASP | 0.452 | 2.04E-09 | NSD2 | 0.408 | 8.82E-08 |
| FANCB | 0.506 | 8.71E-12 | EXOSC9 | 0.451 | 2.25E-09 | ATP23 | 0.407 | 8.96E-08 |
| CIP2A | 0.506 | 8.85E-12 | ZWILCH | 0.45 | 2.32E-09 | GINS2 | 0.407 | 9.11E-08 |
| TCERG1 | 0.505 | 1.03E-11 | CENPO | 0.45 | 2.47E-09 | PNO1 | 0.406 | 1.01E-07 |
| FAM72D | 0.504 | 1.11E-11 | TFAM | 0.45 | 2.45E-09 | SIKE1 | 0.406 | 9.79E-08 |
| PWWP2A | 0.504 | 1.09E-11 | C4orf46 | 0.449 | 2.70E-09 | WDR36 | 0.406 | 9.65E-08 |
| DTYMK | 0.503 | 1.27E-11 | CCDC138 | 0.449 | 2.55E-09 | C1D | 0.406 | 1.02E-07 |
| RBM22 | 0.503 | 1.20E-11 | RPE | 0.449 | 2.53E-09 | MKI67 | 0.404 | 1.14E-07 |
| TTC1 | 0.503 | 1.17E-11 | CCNF | 0.448 | 2.88E-09 | THG1L | 0.404 | 1.14E-07 |
| HNRNPAB | 0.502 | 1.42E-11 | DDIAS | 0.448 | 2.85E-09 | KDELC1 | 0.404 | 1.21E-07 |
| RAD54B | 0.502 | 1.36E-11 | HIST1H2BN | 0.448 | 2.83E-09 | DNMT3B | 0.404 | 1.19E-07 |
| CDC20 | 0.501 | 1.47E-11 | PIMREG | 0.448 | 2.75E-09 | TYW3 | 0.403 | 1.27E-07 |
| SMC4 | 0.5 | 1.68E-11 | RBL1 | 0.447 | 3.15E-09 | TTF2 | 0.403 | 1.23E-07 |
| NOL11 | 0.5 | 1.68E-11 | DIAPH3 | 0.447 | 3.14E-09 | CEBPZ | 0.403 | 1.24E-07 |
| KPNA2 | 0.5 | 1.68E-11 | RFWD3 | 0.447 | 3.15E-09 | TMED9 | 0.403 | 1.23E-07 |
| MND1 | 0.5 | 1.60E-11 | CSE1L | 0.446 | 3.40E-09 | THOC1 | 0.403 | 1.30E-07 |
| HNRNPA2B1 | 0.5 | 1.74E-11 | CKAP5 | 0.445 | 3.65E-09 | U2SURP | 0.403 | 1.30E-07 |
| GTSE1 | 0.5 | 1.65E-11 | C21orf58 | 0.445 | 3.83E-09 | RAN | 0.403 | 1.26E-07 |
| NUSAP1 | 0.5 | 1.69E-11 | PNPT1 | 0.445 | 3.78E-09 | RIOK2 | 0.402 | 1.40E-07 |
| SSB | 0.499 | 1.89E-11 | NEDD1 | 0.444 | 4.04E-09 | ZNF354A | 0.402 | 1.33E-07 |
| NDC1 | 0.498 | 2.03E-11 | DCP2 | 0.444 | 4.05E-09 | NUP62CL | 0.402 | 1.37E-07 |
| MTFR2 | 0.498 | 1.98E-11 | SHLD3 | 0.444 | 4.01E-09 | HSPA9 | 0.402 | 1.42E-07 |
| DSCC1 | 0.498 | 2.04E-11 | TPRKB | 0.443 | 4.28E-09 | MAML1 | 0.402 | 1.35E-07 |
| GEMIN5 | 0.497 | 2.32E-11 | MTBP | 0.443 | 4.33E-09 | COMMD10 | 0.402 | 1.34E-07 |
| CCDC34 | 0.496 | 2.46E-11 | CCSAP | 0.443 | 4.51E-09 | MXD3 | 0.402 | 1.40E-07 |
| CKS2 | 0.496 | 2.64E-11 | HNRNPR | 0.443 | 4.49E-09 | DONSON | 0.402 | 1.43E-07 |
| FANCI | 0.495 | 2.86E-11 | KNL1 | 0.442 | 4.95E-09 | FAM102A | -0.413 | 5.77E-08 |

Note: Note: TCGA, The Cancer Genome Atlas; CorGene, co-expressed gene; Cor, correlation coefficient.

Table S2 Biological function of SPDL1 co-expressed genes.

| Ontology | ID | Description | Count | p.adjust |
| --- | --- | --- | --- | --- |
| BP | GO:0007059 | chromosome segregation | 73 | 6.38E-59 |
| BP | GO:0000280 | nuclear division | 74 | 1.39E-51 |
| BP | GO:0140014 | mitotic nuclear division | 63 | 9.44E-51 |
| BP | GO:0000819 | sister chromatid segregation | 54 | 5.26E-50 |
| BP | GO:0048285 | organelle fission | 75 | 8.67E-50 |
| BP | GO:0098813 | nuclear chromosome segregation | 60 | 3.32E-49 |
| BP | GO:0000070 | mitotic sister chromatid segregation | 49 | 2.74E-47 |
| BP | GO:0000226 | microtubule cytoskeleton organization | 68 | 1.32E-37 |
| BP | GO:0006260 | DNA replication | 50 | 1.09E-32 |
| BP | GO:0051983 | regulation of chromosome segregation | 31 | 1.07E-27 |
| BP | GO:0007088 | regulation of mitotic nuclear division | 36 | 9.76E-27 |
| BP | GO:0007051 | spindle organization | 35 | 7.29E-26 |
| BP | GO:0051783 | regulation of nuclear division | 36 | 1.46E-24 |
| BP | GO:0071103 | DNA conformation change | 43 | 6.43E-24 |
| BP | GO:0000075 | cell cycle checkpoint | 37 | 1.46E-23 |
| BP | GO:1901987 | regulation of cell cycle phase transition | 52 | 1.44E-22 |
| BP | GO:1901990 | regulation of mitotic cell cycle phase transition | 50 | 1.76E-22 |
| BP | GO:0007093 | mitotic cell cycle checkpoint | 32 | 2.12E-22 |
| BP | GO:1902850 | microtubule cytoskeleton organization involved in mitosis | 29 | 4.53E-22 |
| BP | GO:0033045 | regulation of sister chromatid segregation | 24 | 8.46E-22 |
| BP | GO:0006261 | DNA-dependent DNA replication | 30 | 8.48E-22 |
| BP | GO:0007052 | mitotic spindle organization | 26 | 7.33E-21 |
| BP | GO:0008608 | attachment of spindle microtubules to kinetochore | 17 | 2.63E-20 |
| BP | GO:0051052 | regulation of DNA metabolic process | 46 | 2.78E-20 |
| BP | GO:0090068 | positive regulation of cell cycle process | 38 | 4.78E-19 |
| BP | GO:0034508 | centromere complex assembly | 20 | 6.32E-19 |
| BP | GO:0051310 | metaphase plate congression | 20 | 9.40E-19 |
| BP | GO:0033047 | regulation of mitotic sister chromatid segregation | 20 | 2.10E-18 |
| BP | GO:0045787 | positive regulation of cell cycle | 42 | 2.88E-18 |
| BP | GO:0007098 | centrosome cycle | 25 | 8.60E-18 |
| BP | GO:0044839 | cell cycle G2/M phase transition | 35 | 9.25E-18 |
| BP | GO:0050000 | chromosome localization | 21 | 1.64E-17 |
| BP | GO:0051303 | establishment of chromosome localization | 21 | 1.64E-17 |
| BP | GO:0007091 | metaphase/anaphase transition of mitotic cell cycle | 18 | 2.10E-17 |
| BP | GO:0010965 | regulation of mitotic sister chromatid separation | 18 | 2.10E-17 |
| BP | GO:0007080 | mitotic metaphase plate congression | 17 | 3.96E-17 |
| BP | GO:0044784 | metaphase/anaphase transition of cell cycle | 18 | 4.72E-17 |
| BP | GO:0051306 | mitotic sister chromatid separation | 18 | 4.72E-17 |
| BP | GO:0031023 | microtubule organizing center organization | 25 | 6.07E-17 |
| BP | GO:0051304 | chromosome separation | 20 | 8.18E-17 |
| BP | GO:1905818 | regulation of chromosome separation | 18 | 1.53E-16 |
| BP | GO:0070507 | regulation of microtubule cytoskeleton organization | 28 | 2.07E-16 |
| BP | GO:0000082 | G1/S transition of mitotic cell cycle | 34 | 2.44E-16 |
| BP | GO:0044843 | cell cycle G1/S phase transition | 35 | 2.68E-16 |
| BP | GO:0045930 | negative regulation of mitotic cell cycle | 36 | 3.09E-16 |
| BP | GO:0031055 | chromatin remodeling at centromere | 17 | 3.27E-16 |
| BP | GO:0033044 | regulation of chromosome organization | 36 | 4.06E-16 |
| BP | GO:0043486 | histone exchange | 18 | 4.11E-16 |
| BP | GO:0071459 | protein localization to chromosome, centromeric region | 13 | 4.11E-16 |
| BP | GO:0006323 | DNA packaging | 29 | 6.60E-16 |
| BP | GO:0034080 | CENP-A containing nucleosome assembly | 16 | 1.55E-15 |
| BP | GO:0061641 | CENP-A containing chromatin organization | 16 | 1.55E-15 |
| BP | GO:0051321 | meiotic cell cycle | 29 | 2.19E-15 |
| BP | GO:0000086 | G2/M transition of mitotic cell cycle | 31 | 2.57E-15 |
| BP | GO:0030071 | regulation of mitotic metaphase/anaphase transition | 16 | 3.53E-15 |
| BP | GO:0006336 | DNA replication-independent nucleosome assembly | 17 | 4.30E-15 |
| BP | GO:0034724 | DNA replication-independent nucleosome organization | 17 | 4.30E-15 |
| BP | GO:0006310 | DNA recombination | 31 | 4.91E-15 |
| BP | GO:1902099 | regulation of metaphase/anaphase transition of cell cycle | 16 | 7.56E-15 |
| BP | GO:0010948 | negative regulation of cell cycle process | 36 | 8.30E-15 |
| BP | GO:0032886 | regulation of microtubule-based process | 28 | 1.00E-14 |
| BP | GO:0043044 | ATP-dependent chromatin remodeling | 19 | 1.45E-14 |
| BP | GO:1902749 | regulation of cell cycle G2/M phase transition | 28 | 1.45E-14 |
| BP | GO:0031570 | DNA integrity checkpoint | 23 | 1.78E-13 |
| BP | GO:0051225 | spindle assembly | 19 | 3.14E-13 |
| BP | GO:0044786 | cell cycle DNA replication | 16 | 3.25E-13 |
| BP | GO:0006302 | double-strand break repair | 27 | 3.51E-13 |
| BP | GO:0051383 | kinetochore organization | 11 | 6.65E-13 |
| BP | GO:0034502 | protein localization to chromosome | 18 | 7.31E-13 |
| BP | GO:0006338 | chromatin remodeling | 23 | 1.54E-12 |
| BP | GO:0034501 | protein localization to kinetochore | 10 | 1.68E-12 |
| BP | GO:0140013 | meiotic nuclear division | 22 | 1.89E-12 |
| BP | GO:0065004 | protein-DNA complex assembly | 26 | 2.25E-12 |
| BP | GO:0045839 | negative regulation of mitotic nuclear division | 14 | 2.81E-12 |
| BP | GO:0006275 | regulation of DNA replication | 19 | 3.06E-12 |
| BP | GO:1901988 | negative regulation of cell cycle phase transition | 28 | 4.41E-12 |
| BP | GO:1903046 | meiotic cell cycle process | 22 | 6.47E-12 |
| BP | GO:0010389 | regulation of G2/M transition of mitotic cell cycle | 24 | 6.99E-12 |
| BP | GO:0071824 | protein-DNA complex subunit organization | 27 | 7.12E-12 |
| BP | GO:0090329 | regulation of DNA-dependent DNA replication | 14 | 7.12E-12 |
| BP | GO:0006913 | nucleocytoplasmic transport | 30 | 8.58E-12 |
| BP | GO:0051169 | nuclear transport | 30 | 1.10E-11 |
| BP | GO:0045841 | negative regulation of mitotic metaphase/anaphase transition | 12 | 1.13E-11 |
| BP | GO:2000816 | negative regulation of mitotic sister chromatid separation | 12 | 1.13E-11 |
| BP | GO:2001251 | negative regulation of chromosome organization | 20 | 1.21E-11 |
| BP | GO:1902100 | negative regulation of metaphase/anaphase transition of cell cycle | 12 | 1.69E-11 |
| BP | GO:1905819 | negative regulation of chromosome separation | 12 | 1.69E-11 |
| BP | GO:0051784 | negative regulation of nuclear division | 14 | 2.21E-11 |
| BP | GO:1901991 | negative regulation of mitotic cell cycle phase transition | 26 | 2.64E-11 |
| BP | GO:0033048 | negative regulation of mitotic sister chromatid segregation | 12 | 3.71E-11 |
| BP | GO:0000077 | DNA damage checkpoint | 20 | 3.76E-11 |
| BP | GO:0000723 | telomere maintenance | 21 | 7.18E-11 |
| BP | GO:0033046 | negative regulation of sister chromatid segregation | 12 | 7.69E-11 |
| BP | GO:0000724 | double-strand break repair via homologous recombination | 18 | 8.03E-11 |
| BP | GO:0033260 | nuclear DNA replication | 13 | 9.25E-11 |
| BP | GO:0000725 | recombinational repair | 18 | 9.25E-11 |
| BP | GO:0051985 | negative regulation of chromosome segregation | 12 | 1.06E-10 |
| BP | GO:0007094 | mitotic spindle assembly checkpoint | 11 | 1.22E-10 |
| BP | GO:0031577 | spindle checkpoint | 11 | 1.22E-10 |
| BP | GO:0071173 | spindle assembly checkpoint | 11 | 1.22E-10 |
| BP | GO:0071174 | mitotic spindle checkpoint | 11 | 1.22E-10 |
| BP | GO:0007062 | sister chromatid cohesion | 13 | 2.06E-10 |
| BP | GO:0032392 | DNA geometric change | 16 | 2.07E-10 |
| BP | GO:0032200 | telomere organization | 21 | 3.10E-10 |
| BP | GO:0090307 | mitotic spindle assembly | 13 | 3.46E-10 |
| BP | GO:0006334 | nucleosome assembly | 19 | 3.91E-10 |
| BP | GO:0031497 | chromatin assembly | 20 | 4.53E-10 |
| BP | GO:0046605 | regulation of centrosome cycle | 13 | 5.66E-10 |
| BP | GO:0006333 | chromatin assembly or disassembly | 21 | 1.01E-09 |
| BP | GO:0061640 | cytoskeleton-dependent cytokinesis | 15 | 1.12E-09 |
| BP | GO:0000281 | mitotic cytokinesis | 14 | 1.13E-09 |
| BP | GO:0034728 | nucleosome organization | 20 | 1.75E-09 |
| BP | GO:0051315 | attachment of mitotic spindle microtubules to kinetochore | 7 | 2.35E-09 |
| BP | GO:0006403 | RNA localization | 22 | 2.87E-09 |
| BP | GO:0036297 | interstrand cross-link repair | 12 | 3.24E-09 |
| BP | GO:0045931 | positive regulation of mitotic cell cycle | 19 | 3.69E-09 |
| BP | GO:0072331 | signal transduction by p53 class mediator | 24 | 9.37E-09 |
| BP | GO:0051054 | positive regulation of DNA metabolic process | 22 | 9.75E-09 |
| BP | GO:0031145 | anaphase-promoting complex-dependent catabolic process | 14 | 1.01E-08 |
| BP | GO:0000910 | cytokinesis | 18 | 1.22E-08 |
| BP | GO:0051988 | regulation of attachment of spindle microtubules to kinetochore | 7 | 1.40E-08 |
| BP | GO:0051298 | centrosome duplication | 12 | 2.38E-08 |
| BP | GO:0051656 | establishment of organelle localization | 31 | 2.38E-08 |
| BP | GO:0072401 | signal transduction involved in DNA integrity checkpoint | 13 | 2.72E-08 |
| BP | GO:0072422 | signal transduction involved in DNA damage checkpoint | 13 | 2.72E-08 |
| BP | GO:0010639 | negative regulation of organelle organization | 27 | 3.11E-08 |
| BP | GO:0072395 | signal transduction involved in cell cycle checkpoint | 13 | 3.19E-08 |
| BP | GO:0051168 | nuclear export | 19 | 3.32E-08 |
| BP | GO:0044774 | mitotic DNA integrity checkpoint | 14 | 3.93E-08 |
| BP | GO:0032508 | DNA duplex unwinding | 13 | 4.39E-08 |
| BP | GO:0006611 | protein export from nucleus | 18 | 6.04E-08 |
| BP | GO:0030261 | chromosome condensation | 9 | 1.23E-07 |
| BP | GO:0071897 | DNA biosynthetic process | 19 | 1.37E-07 |
| BP | GO:0000018 | regulation of DNA recombination | 13 | 1.51E-07 |
| BP | GO:0022613 | ribonucleoprotein complex biogenesis | 27 | 1.59E-07 |
| BP | GO:0051382 | kinetochore assembly | 7 | 1.68E-07 |
| BP | GO:0031503 | protein-containing complex localization | 21 | 1.69E-07 |
| BP | GO:0050657 | nucleic acid transport | 18 | 1.93E-07 |
| BP | GO:0050658 | RNA transport | 18 | 1.93E-07 |
| BP | GO:0044773 | mitotic DNA damage checkpoint | 13 | 1.95E-07 |
| BP | GO:0051236 | establishment of RNA localization | 18 | 2.49E-07 |
| BP | GO:0006405 | RNA export from nucleus | 15 | 2.64E-07 |
| BP | GO:0051493 | regulation of cytoskeleton organization | 30 | 2.69E-07 |
| BP | GO:0051053 | negative regulation of DNA metabolic process | 16 | 2.77E-07 |
| BP | GO:0006890 | retrograde vesicle-mediated transport, Golgi to ER | 12 | 4.15E-07 |
| BP | GO:0032201 | telomere maintenance via semi-conservative replication | 8 | 4.89E-07 |
| BP | GO:1902751 | positive regulation of cell cycle G2/M phase transition | 8 | 4.89E-07 |
| BP | GO:0016572 | histone phosphorylation | 9 | 5.74E-07 |
| BP | GO:1901989 | positive regulation of cell cycle phase transition | 13 | 8.06E-07 |
| BP | GO:0000083 | regulation of transcription involved in G1/S transition of mitotic cell cycle | 8 | 8.94E-07 |
| BP | GO:0010824 | regulation of centrosome duplication | 9 | 9.11E-07 |
| BP | GO:0071426 | ribonucleoprotein complex export from nucleus | 14 | 9.41E-07 |
| BP | GO:0045132 | meiotic chromosome segregation | 11 | 9.90E-07 |
| BP | GO:0071166 | ribonucleoprotein complex localization | 14 | 1.04E-06 |
| BP | GO:0090224 | regulation of spindle organization | 9 | 1.12E-06 |
| BP | GO:0006270 | DNA replication initiation | 8 | 1.15E-06 |
| BP | GO:0031572 | G2 DNA damage checkpoint | 8 | 1.15E-06 |
| BP | GO:1901796 | regulation of signal transduction by p53 class mediator | 17 | 1.30E-06 |
| BP | GO:2001020 | regulation of response to DNA damage stimulus | 18 | 1.33E-06 |
| BP | GO:0051302 | regulation of cell division | 16 | 1.78E-06 |
| BP | GO:2000779 | regulation of double-strand break repair | 11 | 2.03E-06 |
| BP | GO:0007064 | mitotic sister chromatid cohesion | 7 | 2.68E-06 |
| BP | GO:0006397 | mRNA processing | 29 | 2.80E-06 |
| BP | GO:0007076 | mitotic chromosome condensation | 6 | 3.16E-06 |
| BP | GO:0042770 | signal transduction in response to DNA damage | 14 | 4.00E-06 |
| BP | GO:0045740 | positive regulation of DNA replication | 8 | 4.00E-06 |
| BP | GO:0006282 | regulation of DNA repair | 13 | 4.02E-06 |
| BP | GO:0031109 | microtubule polymerization or depolymerization | 12 | 4.02E-06 |
| BP | GO:0000377 | RNA splicing, via transesterification reactions with bulged adenosine as nucleophile | 23 | 4.56E-06 |
| BP | GO:0000398 | mRNA splicing, via spliceosome | 23 | 4.56E-06 |
| BP | GO:0022616 | DNA strand elongation | 6 | 4.77E-06 |
| BP | GO:0031571 | mitotic G1 DNA damage checkpoint | 10 | 4.83E-06 |
| BP | GO:0044819 | mitotic G1/S transition checkpoint | 10 | 4.83E-06 |
| BP | GO:0015931 | nucleobase-containing compound transport | 18 | 5.04E-06 |
| BP | GO:0000375 | RNA splicing, via transesterification reactions | 23 | 5.43E-06 |
| BP | GO:0019886 | antigen processing and presentation of exogenous peptide antigen via MHC class II | 12 | 5.43E-06 |
| BP | GO:0044783 | G1 DNA damage checkpoint | 10 | 5.50E-06 |
| BP | GO:0060236 | regulation of mitotic spindle organization | 8 | 5.91E-06 |
| BP | GO:1901992 | positive regulation of mitotic cell cycle phase transition | 11 | 7.09E-06 |
| BP | GO:0002495 | antigen processing and presentation of peptide antigen via MHC class II | 12 | 7.42E-06 |
| BP | GO:0032465 | regulation of cytokinesis | 11 | 7.92E-06 |
| BP | GO:0051231 | spindle elongation | 5 | 7.92E-06 |
| BP | GO:0008380 | RNA splicing | 26 | 7.92E-06 |
| BP | GO:0002504 | antigen processing and presentation of peptide or polysaccharide antigen via MHC class II | 12 | 8.10E-06 |
| BP | GO:0007050 | cell cycle arrest | 18 | 9.08E-06 |
| BP | GO:2000278 | regulation of DNA biosynthetic process | 12 | 1.35E-05 |
| BP | GO:0000076 | DNA replication checkpoint | 5 | 1.39E-05 |
| BP | GO:2000105 | positive regulation of DNA-dependent DNA replication | 5 | 1.39E-05 |
| BP | GO:0006977 | DNA damage response, signal transduction by p53 class mediator resulting in cell cycle arrest | 9 | 1.53E-05 |
| BP | GO:0007143 | female meiotic nuclear division | 7 | 1.69E-05 |
| BP | GO:0072431 | signal transduction involved in mitotic G1 DNA damage checkpoint | 9 | 1.76E-05 |
| BP | GO:1902400 | intracellular signal transduction involved in G1 DNA damage checkpoint | 9 | 1.76E-05 |
| BP | GO:0071826 | ribonucleoprotein complex subunit organization | 17 | 2.09E-05 |
| BP | GO:0007099 | centriole replication | 7 | 2.09E-05 |
| BP | GO:1901976 | regulation of cell cycle checkpoint | 7 | 2.09E-05 |
| BP | GO:0072413 | signal transduction involved in mitotic cell cycle checkpoint | 9 | 2.30E-05 |
| BP | GO:1902402 | signal transduction involved in mitotic DNA damage checkpoint | 9 | 2.30E-05 |
| BP | GO:1902403 | signal transduction involved in mitotic DNA integrity checkpoint | 9 | 2.30E-05 |
| BP | GO:0045005 | DNA-dependent DNA replication maintenance of fidelity | 7 | 3.22E-05 |
| BP | GO:1902750 | negative regulation of cell cycle G2/M phase transition | 11 | 3.47E-05 |
| BP | GO:0010833 | telomere maintenance via telomere lengthening | 10 | 3.55E-05 |
| BP | GO:0007019 | microtubule depolymerization | 7 | 3.92E-05 |
| BP | GO:0045840 | positive regulation of mitotic nuclear division | 8 | 4.20E-05 |
| BP | GO:0044818 | mitotic G2/M transition checkpoint | 6 | 4.49E-05 |
| BP | GO:0098534 | centriole assembly | 7 | 4.75E-05 |
| BP | GO:0071158 | positive regulation of cell cycle arrest | 10 | 4.88E-05 |
| BP | GO:0090231 | regulation of spindle checkpoint | 5 | 5.14E-05 |
| BP | GO:0090266 | regulation of mitotic cell cycle spindle assembly checkpoint | 5 | 5.14E-05 |
| BP | GO:1903504 | regulation of mitotic spindle checkpoint | 5 | 5.14E-05 |
| BP | GO:0010971 | positive regulation of G2/M transition of mitotic cell cycle | 6 | 5.69E-05 |
| BP | GO:0051984 | positive regulation of chromosome segregation | 6 | 5.69E-05 |
| BP | GO:0002200 | somatic diversification of immune receptors | 9 | 6.36E-05 |
| BP | GO:0032467 | positive regulation of cytokinesis | 7 | 6.74E-05 |
| BP | GO:0032506 | cytokinetic process | 7 | 6.74E-05 |
| BP | GO:0060249 | anatomical structure homeostasis | 23 | 8.02E-05 |
| BP | GO:0043161 | proteasome-mediated ubiquitin-dependent protein catabolic process | 20 | 8.43E-05 |
| BP | GO:0007127 | meiosis I | 10 | 8.81E-05 |
| BP | GO:0007018 | microtubule-based movement | 16 | 9.66E-05 |
| BP | GO:0007096 | regulation of exit from mitosis | 5 | 0.000103708 |
| BP | GO:0016445 | somatic diversification of immunoglobulins | 8 | 0.000110828 |
| BP | GO:0061982 | meiosis I cell cycle process | 10 | 0.000116859 |
| BP | GO:0010569 | regulation of double-strand break repair via homologous recombination | 7 | 0.000131933 |
| BP | GO:0051653 | spindle localization | 7 | 0.000131933 |
| BP | GO:0040001 | establishment of mitotic spindle localization | 6 | 0.00014048 |
| BP | GO:0022618 | ribonucleoprotein complex assembly | 15 | 0.000167654 |
| BP | GO:0031297 | replication fork processing | 6 | 0.000172672 |
| BP | GO:0051782 | negative regulation of cell division | 5 | 0.000190003 |
| BP | GO:0002562 | somatic diversification of immune receptors via germline recombination within a single locus | 8 | 0.000204547 |
| BP | GO:0016444 | somatic cell DNA recombination | 8 | 0.000204547 |
| BP | GO:0051785 | positive regulation of nuclear division | 8 | 0.000204547 |
| BP | GO:0090305 | nucleic acid phosphodiester bond hydrolysis | 17 | 0.000227201 |
| BP | GO:1904356 | regulation of telomere maintenance via telomere lengthening | 8 | 0.000229445 |
| BP | GO:0022412 | cellular process involved in reproduction in multicellular organism | 19 | 0.00024613 |
| BP | GO:0045910 | negative regulation of DNA recombination | 6 | 0.000250062 |
| BP | GO:0010212 | response to ionizing radiation | 12 | 0.000259102 |
| BP | GO:0051255 | spindle midzone assembly | 4 | 0.000259188 |
| BP | GO:2001252 | positive regulation of chromosome organization | 13 | 0.000280529 |
| BP | GO:1903311 | regulation of mRNA metabolic process | 17 | 0.000305987 |
| BP | GO:0002204 | somatic recombination of immunoglobulin genes involved in immune response | 7 | 0.000306121 |
| BP | GO:0002208 | somatic diversification of immunoglobulins involved in immune response | 7 | 0.000306121 |
| BP | GO:0006301 | postreplication repair | 7 | 0.000306121 |
| BP | GO:0045190 | isotype switching | 7 | 0.000306121 |
| BP | GO:0072698 | protein localization to microtubule cytoskeleton | 7 | 0.000306121 |
| BP | GO:0051170 | import into nucleus | 11 | 0.000324862 |
| BP | GO:0007100 | mitotic centrosome separation | 4 | 0.000384687 |
| BP | GO:0035404 | histone-serine phosphorylation | 4 | 0.000384687 |
| BP | GO:2000573 | positive regulation of DNA biosynthetic process | 8 | 0.000385987 |
| BP | GO:2000104 | negative regulation of DNA-dependent DNA replication | 5 | 0.000393475 |
| BP | GO:0016447 | somatic recombination of immunoglobulin gene segments | 7 | 0.000394531 |
| BP | GO:0030330 | DNA damage response, signal transduction by p53 class mediator | 10 | 0.000394741 |
| BP | GO:0007004 | telomere maintenance via telomerase | 8 | 0.000421219 |
| BP | GO:0006406 | mRNA export from nucleus | 10 | 0.000421219 |
| BP | GO:0031123 | RNA 3'-end processing | 10 | 0.000421219 |
| BP | GO:0071427 | mRNA-containing ribonucleoprotein complex export from nucleus | 10 | 0.000421219 |
| BP | GO:0044380 | protein localization to cytoskeleton | 7 | 0.000440487 |
| BP | GO:0071156 | regulation of cell cycle arrest | 10 | 0.000452667 |
| BP | GO:1904666 | regulation of ubiquitin protein ligase activity | 5 | 0.000483492 |
| BP | GO:1903312 | negative regulation of mRNA metabolic process | 8 | 0.00051116 |
| BP | GO:0031124 | mRNA 3'-end processing | 9 | 0.000530069 |
| BP | GO:0007077 | mitotic nuclear envelope disassembly | 4 | 0.00053545 |
| BP | GO:0051299 | centrosome separation | 4 | 0.00053545 |
| BP | GO:0008156 | negative regulation of DNA replication | 6 | 0.00053977 |
| BP | GO:0051293 | establishment of spindle localization | 6 | 0.00053977 |
| BP | GO:0097064 | ncRNA export from nucleus | 6 | 0.00053977 |
| BP | GO:0070192 | chromosome organization involved in meiotic cell cycle | 7 | 0.000548939 |
| BP | GO:0000729 | DNA double-strand break processing | 5 | 0.000587325 |
| BP | GO:0010498 | proteasomal protein catabolic process | 20 | 0.000598146 |
| BP | GO:0032210 | regulation of telomere maintenance via telomerase | 7 | 0.000614965 |
| BP | GO:0009411 | response to UV | 11 | 0.000682627 |
| BP | GO:0042769 | DNA damage response, detection of DNA damage | 6 | 0.000723702 |
| BP | GO:0044766 | multi-organism transport | 8 | 0.000727994 |
| BP | GO:1902579 | multi-organism localization | 8 | 0.000727994 |
| BP | GO:0062033 | positive regulation of mitotic sister chromatid segregation | 4 | 0.000727994 |
| BP | GO:0002381 | immunoglobulin production involved in immunoglobulin mediated immune response | 7 | 0.000765689 |
| BP | GO:0006278 | RNA-dependent DNA biosynthetic process | 8 | 0.000791017 |
| BP | GO:1900182 | positive regulation of protein localization to nucleus | 8 | 0.000791017 |
| BP | GO:0075733 | intracellular transport of virus | 7 | 0.000954175 |
| BP | GO:0046606 | negative regulation of centrosome cycle | 4 | 0.000981857 |
| BP | GO:0072425 | signal transduction involved in G2 DNA damage checkpoint | 4 | 0.000981857 |
| BP | GO:0070317 | negative regulation of G0 to G1 transition | 6 | 0.00108165 |
| BP | GO:0051028 | mRNA transport | 11 | 0.001154818 |
| BP | GO:0009314 | response to radiation | 21 | 0.001174829 |
| BP | GO:0032204 | regulation of telomere maintenance | 8 | 0.001222248 |
| BP | GO:2001021 | negative regulation of response to DNA damage stimulus | 8 | 0.001222248 |
| BP | GO:0050686 | negative regulation of mRNA processing | 5 | 0.001228627 |
| BP | GO:0090169 | regulation of spindle assembly | 5 | 0.001228627 |
| BP | GO:0046794 | transport of virus | 7 | 0.00127919 |
| BP | GO:2000134 | negative regulation of G1/S transition of mitotic cell cycle | 10 | 0.001285681 |
| BP | GO:0002478 | antigen processing and presentation of exogenous peptide antigen | 12 | 0.001312539 |
| BP | GO:0042254 | ribosome biogenesis | 13 | 0.001412869 |
| BP | GO:0010458 | exit from mitosis | 5 | 0.00144538 |
| BP | GO:0019884 | antigen processing and presentation of exogenous antigen | 12 | 0.00144538 |
| BP | GO:2000045 | regulation of G1/S transition of mitotic cell cycle | 12 | 0.001518467 |
| BP | GO:0030397 | membrane disassembly | 4 | 0.001636624 |
| BP | GO:0051081 | nuclear envelope disassembly | 4 | 0.001636624 |
| BP | GO:0000079 | regulation of cyclin-dependent protein serine/threonine kinase activity | 8 | 0.00165654 |
| BP | GO:0070316 | regulation of G0 to G1 transition | 6 | 0.001729231 |
| BP | GO:1902807 | negative regulation of cell cycle G1/S phase transition | 10 | 0.001828899 |
| BP | GO:0010972 | negative regulation of G2/M transition of mitotic cell cycle | 8 | 0.001934083 |
| BP | GO:0007292 | female gamete generation | 10 | 0.00205587 |
| BP | GO:0034629 | cellular protein-containing complex localization | 4 | 0.002065177 |
| BP | GO:0045023 | G0 to G1 transition | 6 | 0.002169186 |
| BP | GO:1904029 | regulation of cyclin-dependent protein kinase activity | 8 | 0.002240088 |
| BP | GO:0051972 | regulation of telomerase activity | 6 | 0.002422519 |
| BP | GO:0048002 | antigen processing and presentation of peptide antigen | 12 | 0.002539488 |
| BP | GO:0000387 | spliceosomal snRNP assembly | 6 | 0.003015519 |
| BP | GO:0006409 | tRNA export from nucleus | 5 | 0.00302549 |
| BP | GO:0045191 | regulation of isotype switching | 5 | 0.00302549 |
| BP | GO:0051031 | tRNA transport | 5 | 0.00302549 |
| BP | GO:0071431 | tRNA-containing ribonucleoprotein complex export from nucleus | 5 | 0.00302549 |
| BP | GO:0007095 | mitotic G2 DNA damage checkpoint | 4 | 0.003135406 |
| BP | GO:0046599 | regulation of centriole replication | 4 | 0.003135406 |
| BP | GO:0048025 | negative regulation of mRNA splicing, via spliceosome | 4 | 0.003135406 |
| BP | GO:0002312 | B cell activation involved in immune response | 7 | 0.003348427 |
| BP | GO:0034504 | protein localization to nucleus | 13 | 0.003383253 |
| BP | GO:0043624 | cellular protein complex disassembly | 12 | 0.003398083 |
| BP | GO:1902806 | regulation of cell cycle G1/S phase transition | 12 | 0.003398083 |
| BP | GO:0007131 | reciprocal meiotic recombination | 5 | 0.003402749 |
| BP | GO:0032211 | negative regulation of telomere maintenance via telomerase | 4 | 0.003774492 |
| BP | GO:1901673 | regulation of mitotic spindle assembly | 4 | 0.003774492 |
| BP | GO:0097711 | ciliary basal body-plasma membrane docking | 8 | 0.003840862 |
| BP | GO:0035825 | homologous recombination | 5 | 0.003858068 |
| BP | GO:0045911 | positive regulation of DNA recombination | 5 | 0.004381743 |
| BP | GO:0051973 | positive regulation of telomerase activity | 5 | 0.004381743 |
| BP | GO:0007063 | regulation of sister chromatid cohesion | 4 | 0.004520228 |
| BP | GO:0002377 | immunoglobulin production | 8 | 0.004653957 |
| BP | GO:0022411 | cellular component disassembly | 21 | 0.005166814 |
| BP | GO:0009263 | deoxyribonucleotide biosynthetic process | 3 | 0.005387392 |
| BP | GO:0042023 | DNA endoreduplication | 3 | 0.005387392 |
| BP | GO:0070601 | centromeric sister chromatid cohesion | 3 | 0.005387392 |
| BP | GO:0006284 | base-excision repair | 5 | 0.005530268 |
| BP | GO:0019985 | translesion synthesis | 5 | 0.006985496 |
| BP | GO:0006303 | double-strand break repair via nonhomologous end joining | 7 | 0.006985496 |
| BP | GO:0016446 | somatic hypermutation of immunoglobulin genes | 3 | 0.0072127 |
| BP | GO:1902115 | regulation of organelle assembly | 11 | 0.007237302 |
| BP | GO:0046824 | positive regulation of nucleocytoplasmic transport | 6 | 0.007341295 |
| BP | GO:0000132 | establishment of mitotic spindle orientation | 4 | 0.007366377 |
| BP | GO:0033119 | negative regulation of RNA splicing | 4 | 0.007366377 |
| BP | GO:0050684 | regulation of mRNA processing | 8 | 0.008455251 |
| BP | GO:0051781 | positive regulation of cell division | 7 | 0.008477096 |
| BP | GO:0001556 | oocyte maturation | 4 | 0.008509029 |
| BP | GO:0006730 | one-carbon metabolic process | 4 | 0.008509029 |
| BP | GO:0045830 | positive regulation of isotype switching | 4 | 0.008509029 |
| BP | GO:0019882 | antigen processing and presentation | 12 | 0.008972401 |
| BP | GO:0000212 | meiotic spindle organization | 3 | 0.009044204 |
| BP | GO:0000460 | maturation of 5.8S rRNA | 3 | 0.009044204 |
| BP | GO:0002566 | somatic diversification of immune receptors via somatic mutation | 3 | 0.009044204 |
| BP | GO:0010457 | centriole-centriole cohesion | 3 | 0.009044204 |
| BP | GO:0010826 | negative regulation of centrosome duplication | 3 | 0.009044204 |
| BP | GO:1904668 | positive regulation of ubiquitin protein ligase activity | 3 | 0.009044204 |
| BP | GO:0031398 | positive regulation of protein ubiquitination | 8 | 0.009188896 |
| BP | GO:0031396 | regulation of protein ubiquitination | 11 | 0.010939656 |
| BP | GO:1904357 | negative regulation of telomere maintenance via telomere lengthening | 4 | 0.011121544 |
| BP | GO:0000726 | non-recombinational repair | 7 | 0.011391826 |
| BP | GO:0032988 | ribonucleoprotein complex disassembly | 3 | 0.011391826 |
| BP | GO:0048291 | isotype switching to IgG isotypes | 3 | 0.011391826 |
| BP | GO:0034470 | ncRNA processing | 14 | 0.01256286 |
| BP | GO:0010165 | response to X-ray | 4 | 0.01256286 |
| BP | GO:0051294 | establishment of spindle orientation | 4 | 0.01256286 |
| BP | GO:1900180 | regulation of protein localization to nucleus | 8 | 0.013252744 |
| BP | GO:0051438 | regulation of ubiquitin-protein transferase activity | 5 | 0.013417292 |
| BP | GO:0006997 | nucleus organization | 8 | 0.013902031 |
| BP | GO:0035372 | protein localization to microtubule | 3 | 0.013965153 |
| BP | GO:0051307 | meiotic chromosome separation | 3 | 0.013965153 |
| BP | GO:0031110 | regulation of microtubule polymerization or depolymerization | 6 | 0.013965153 |
| BP | GO:0051261 | protein depolymerization | 7 | 0.01418014 |
| BP | GO:0032984 | protein-containing complex disassembly | 14 | 0.014311565 |
| BP | GO:0034660 | ncRNA metabolic process | 19 | 0.014912949 |
| BP | GO:2000780 | negative regulation of double-strand break repair | 4 | 0.015862404 |
| BP | GO:0000245 | spliceosomal complex assembly | 6 | 0.01592983 |
| BP | GO:0007281 | germ cell development | 12 | 0.016016297 |
| BP | GO:0061013 | regulation of mRNA catabolic process | 10 | 0.016391136 |
| BP | GO:0001325 | formation of extrachromosomal circular DNA | 3 | 0.016710887 |
| BP | GO:0090656 | t-circle formation | 3 | 0.016710887 |
| BP | GO:0090737 | telomere maintenance via telomere trimming | 3 | 0.016710887 |
| BP | GO:0000731 | DNA synthesis involved in DNA repair | 5 | 0.016961663 |
| BP | GO:0002712 | regulation of B cell mediated immunity | 5 | 0.018410011 |
| BP | GO:0002889 | regulation of immunoglobulin mediated immune response | 5 | 0.018410011 |
| BP | GO:0045738 | negative regulation of DNA repair | 4 | 0.019650382 |
| BP | GO:0006364 | rRNA processing | 9 | 0.019814701 |
| BP | GO:0033262 | regulation of nuclear cell cycle DNA replication | 3 | 0.019898694 |
| BP | GO:0051131 | chaperone-mediated protein complex assembly | 3 | 0.019898694 |
| BP | GO:0031100 | animal organ regeneration | 6 | 0.020216669 |
| BP | GO:0048024 | regulation of mRNA splicing, via spliceosome | 6 | 0.020216669 |
| BP | GO:0046822 | regulation of nucleocytoplasmic transport | 7 | 0.020279097 |
| BP | GO:1903322 | positive regulation of protein modification by small protein conjugation or removal | 8 | 0.021538501 |
| BP | GO:0043928 | exonucleolytic nuclear-transcribed mRNA catabolic process involved in deadenylation-dependent decay | 4 | 0.021564262 |
| BP | GO:0043488 | regulation of mRNA stability | 9 | 0.022981466 |
| BP | GO:0097329 | response to antimetabolite | 3 | 0.023325506 |
| BP | GO:2000042 | negative regulation of double-strand break repair via homologous recombination | 3 | 0.023325506 |
| BP | GO:0000291 | nuclear-transcribed mRNA catabolic process, exonucleolytic | 4 | 0.023820483 |
| BP | GO:1903829 | positive regulation of cellular protein localization | 14 | 0.023933358 |
| BP | GO:1900034 | regulation of cellular response to heat | 6 | 0.025527538 |
| BP | GO:0016072 | rRNA metabolic process | 10 | 0.025527538 |
| BP | GO:1903320 | regulation of protein modification by small protein conjugation or removal | 11 | 0.026229998 |
| BP | GO:0042276 | error-prone translesion synthesis | 3 | 0.027169082 |
| BP | GO:0043487 | regulation of RNA stability | 9 | 0.027514267 |
| BP | GO:0032205 | negative regulation of telomere maintenance | 4 | 0.028752495 |
| BP | GO:0046825 | regulation of protein export from nucleus | 4 | 0.028752495 |
| BP | GO:0031061 | negative regulation of histone methylation | 3 | 0.031276588 |
| BP | GO:0070987 | error-free translesion synthesis | 3 | 0.031276588 |
| BP | GO:0002714 | positive regulation of B cell mediated immunity | 4 | 0.031276588 |
| BP | GO:0002891 | positive regulation of immunoglobulin mediated immune response | 4 | 0.031276588 |
| BP | GO:0042307 | positive regulation of protein import into nucleus | 4 | 0.031276588 |
| BP | GO:1904358 | positive regulation of telomere maintenance via telomere lengthening | 4 | 0.031276588 |
| BP | GO:0048477 | oogenesis | 6 | 0.033340991 |
| BP | GO:2000279 | negative regulation of DNA biosynthetic process | 4 | 0.034255283 |
| BP | GO:0006401 | RNA catabolic process | 15 | 0.034259238 |
| BP | GO:0000966 | RNA 5'-end processing | 3 | 0.035699917 |
| BP | GO:0140056 | organelle localization by membrane tethering | 8 | 0.036106444 |
| BP | GO:1904591 | positive regulation of protein import | 4 | 0.037224114 |
| BP | GO:0071478 | cellular response to radiation | 9 | 0.038768679 |
| BP | GO:0048193 | Golgi vesicle transport | 13 | 0.038878614 |
| BP | GO:0002637 | regulation of immunoglobulin production | 5 | 0.038878614 |
| BP | GO:0043484 | regulation of RNA splicing | 7 | 0.040179664 |
| BP | GO:0046827 | positive regulation of protein export from nucleus | 3 | 0.040360492 |
| BP | GO:0034605 | cellular response to heat | 7 | 0.043705806 |
| BP | GO:0002639 | positive regulation of immunoglobulin production | 4 | 0.043705806 |
| BP | GO:2001032 | regulation of double-strand break repair via nonhomologous end joining | 3 | 0.045732378 |
| BP | GO:0140053 | mitochondrial gene expression | 8 | 0.046653853 |
| CC | GO:0098687 | chromosomal region | 81 | 5.55E-62 |
| CC | GO:0000775 | chromosome, centromeric region | 62 | 7.48E-57 |
| CC | GO:0000776 | kinetochore | 52 | 5.11E-52 |
| CC | GO:0000793 | condensed chromosome | 58 | 2.67E-49 |
| CC | GO:0000779 | condensed chromosome, centromeric region | 46 | 2.61E-47 |
| CC | GO:0000777 | condensed chromosome kinetochore | 43 | 7.28E-45 |
| CC | GO:0005819 | spindle | 60 | 1.38E-38 |
| CC | GO:0005813 | centrosome | 55 | 9.76E-25 |
| CC | GO:0000922 | spindle pole | 32 | 4.27E-22 |
| CC | GO:0005876 | spindle microtubule | 19 | 8.03E-18 |
| CC | GO:0072686 | mitotic spindle | 23 | 4.34E-17 |
| CC | GO:0000940 | condensed chromosome outer kinetochore | 10 | 1.76E-14 |
| CC | GO:0030496 | midbody | 26 | 2.29E-14 |
| CC | GO:0005874 | microtubule | 33 | 7.33E-12 |
| CC | GO:0044454 | nuclear chromosome part | 37 | 3.01E-11 |
| CC | GO:0044450 | microtubule organizing center part | 22 | 9.13E-11 |
| CC | GO:0000781 | chromosome, telomeric region | 19 | 7.39E-09 |
| CC | GO:0005814 | centriole | 17 | 9.29E-09 |
| CC | GO:0000780 | condensed nuclear chromosome, centromeric region | 7 | 3.76E-07 |
| CC | GO:0000794 | condensed nuclear chromosome | 12 | 5.29E-07 |
| CC | GO:0071013 | catalytic step 2 spliceosome | 12 | 1.41E-06 |
| CC | GO:0000785 | chromatin | 30 | 1.67E-06 |
| CC | GO:0051233 | spindle midzone | 7 | 1.19E-05 |
| CC | GO:0005681 | spliceosomal complex | 15 | 1.23E-05 |
| CC | GO:0000784 | nuclear chromosome, telomeric region | 12 | 8.28E-05 |
| CC | GO:0097431 | mitotic spindle pole | 6 | 0.00013405 |
| CC | GO:1990752 | microtubule end | 6 | 0.00013405 |
| CC | GO:0035371 | microtubule plus-end | 5 | 0.000144936 |
| CC | GO:0034719 | SMN-Sm protein complex | 5 | 0.000194988 |
| CC | GO:0005643 | nuclear pore | 8 | 0.000251659 |
| CC | GO:0045171 | intercellular bridge | 8 | 0.000251659 |
| CC | GO:1990023 | mitotic spindle midzone | 4 | 0.000517644 |
| CC | GO:0032797 | SMN complex | 4 | 0.000686392 |
| CC | GO:0097504 | Gemini of coiled bodies | 4 | 0.000686392 |
| CC | GO:0018995 | host | 8 | 0.000686392 |
| CC | GO:0043657 | host cell | 8 | 0.000686392 |
| CC | GO:0005657 | replication fork | 7 | 0.000908104 |
| CC | GO:0043240 | Fanconi anaemia nuclear complex | 4 | 0.000908104 |
| CC | GO:0000152 | nuclear ubiquitin ligase complex | 6 | 0.000908104 |
| CC | GO:0015030 | Cajal body | 8 | 0.001044511 |
| CC | GO:0044215 | other organism | 8 | 0.001044511 |
| CC | GO:0044216 | other organism cell | 8 | 0.001044511 |
| CC | GO:0044217 | other organism part | 8 | 0.001044511 |
| CC | GO:0032153 | cell division site | 7 | 0.001080945 |
| CC | GO:0032155 | cell division site part | 7 | 0.001080945 |
| CC | GO:0034399 | nuclear periphery | 10 | 0.001760185 |
| CC | GO:0016363 | nuclear matrix | 9 | 0.002129518 |
| CC | GO:0090734 | site of DNA damage | 7 | 0.002326265 |
| CC | GO:0005680 | anaphase-promoting complex | 4 | 0.005229654 |
| CC | GO:0032154 | cleavage furrow | 6 | 0.005535927 |
| CC | GO:0016607 | nuclear speck | 18 | 0.005535927 |
| CC | GO:0000176 | nuclear exosome (RNase complex) | 3 | 0.005535927 |
| CC | GO:0000235 | astral microtubule | 3 | 0.005535927 |
| CC | GO:0005818 | aster | 3 | 0.005535927 |
| CC | GO:0072687 | meiotic spindle | 3 | 0.005535927 |
| CC | GO:0005635 | nuclear envelope | 19 | 0.005608267 |
| CC | GO:0042555 | MCM complex | 3 | 0.007235837 |
| CC | GO:0000178 | exosome (RNase complex) | 4 | 0.007353511 |
| CC | GO:1905354 | exoribonuclease complex | 4 | 0.007353511 |
| CC | GO:0005881 | cytoplasmic microtubule | 6 | 0.008776411 |
| CC | GO:0008278 | cohesin complex | 3 | 0.008881515 |
| CC | GO:0031616 | spindle pole centrosome | 3 | 0.011191533 |
| CC | GO:0044815 | DNA packaging complex | 8 | 0.011199544 |
| CC | GO:0090543 | Flemming body | 4 | 0.012262624 |
| CC | GO:0000800 | lateral element | 3 | 0.013182578 |
| CC | GO:0010369 | chromocenter | 3 | 0.013182578 |
| CC | GO:0000307 | cyclin-dependent protein kinase holoenzyme complex | 4 | 0.013375167 |
| CC | GO:0045120 | pronucleus | 3 | 0.015757221 |
| CC | GO:0120114 | Sm-like protein family complex | 7 | 0.016329082 |
| CC | GO:0031461 | cullin-RING ubiquitin ligase complex | 8 | 0.017946021 |
| CC | GO:0000792 | heterochromatin | 6 | 0.019550489 |
| CC | GO:0000790 | nuclear chromatin | 14 | 0.01973549 |
| CC | GO:0005720 | nuclear heterochromatin | 4 | 0.019859297 |
| CC | GO:0000795 | synaptonemal complex | 4 | 0.021565529 |
| CC | GO:0099086 | synaptonemal structure | 4 | 0.021565529 |
| CC | GO:0000930 | gamma-tubulin complex | 3 | 0.024182233 |
| CC | GO:0000151 | ubiquitin ligase complex | 11 | 0.026249379 |
| CC | GO:0005697 | telomerase holoenzyme complex | 3 | 0.027219333 |
| CC | GO:0043073 | germ cell nucleus | 3 | 0.027219333 |
| CC | GO:0005721 | pericentric heterochromatin | 3 | 0.031157553 |
| MF | GO:0008094 | DNA-dependent ATPase activity | 18 | 2.95E-12 |
| MF | GO:0042623 | ATPase activity, coupled | 24 | 7.74E-09 |
| MF | GO:0016887 | ATPase activity | 27 | 7.74E-09 |
| MF | GO:0140097 | catalytic activity, acting on DNA | 20 | 7.74E-09 |
| MF | GO:0004386 | helicase activity | 17 | 1.42E-07 |
| MF | GO:0003697 | single-stranded DNA binding | 14 | 4.04E-07 |
| MF | GO:0003678 | DNA helicase activity | 10 | 7.29E-07 |
| MF | GO:0008017 | microtubule binding | 18 | 7.89E-07 |
| MF | GO:0003682 | chromatin binding | 30 | 7.93E-07 |
| MF | GO:0015631 | tubulin binding | 22 | 9.14E-07 |
| MF | GO:0043142 | single-stranded DNA-dependent ATPase activity | 6 | 2.52E-06 |
| MF | GO:0004003 | ATP-dependent DNA helicase activity | 8 | 6.27E-06 |
| MF | GO:0035173 | histone kinase activity | 6 | 6.67E-06 |
| MF | GO:0000217 | DNA secondary structure binding | 7 | 9.14E-06 |
| MF | GO:0003684 | damaged DNA binding | 10 | 1.24E-05 |
| MF | GO:0008026 | ATP-dependent helicase activity | 9 | 2.95E-05 |
| MF | GO:0070035 | purine NTP-dependent helicase activity | 9 | 2.95E-05 |
| MF | GO:0000400 | four-way junction DNA binding | 5 | 0.00031709 |
| MF | GO:0003777 | microtubule motor activity | 7 | 0.000612839 |
| MF | GO:1990825 | sequence-specific mRNA binding | 4 | 0.001547176 |
| MF | GO:1990939 | ATP-dependent microtubule motor activity | 4 | 0.001547176 |
| MF | GO:0008409 | 5'-3' exonuclease activity | 4 | 0.005074787 |
| MF | GO:0016796 | exonuclease activity, active with either ribo- or deoxyribonucleic acids and producing 5'-phosphomonoesters | 6 | 0.005164449 |
| MF | GO:0003774 | motor activity | 9 | 0.005483923 |
| MF | GO:0051082 | unfolded protein binding | 8 | 0.005741641 |
| MF | GO:0003730 | mRNA 3'-UTR binding | 7 | 0.007511752 |
| MF | GO:0004527 | exonuclease activity | 7 | 0.007511752 |
| MF | GO:0016888 | endodeoxyribonuclease activity, producing 5'-phosphomonoesters | 3 | 0.013043105 |
| MF | GO:0097617 | annealing activity | 3 | 0.013043105 |
| MF | GO:0004674 | protein serine/threonine kinase activity | 18 | 0.013043105 |
| MF | GO:0008187 | poly-pyrimidine tract binding | 4 | 0.013043105 |
| MF | GO:0004518 | nuclease activity | 11 | 0.013043105 |
| MF | GO:0140142 | nucleocytoplasmic carrier activity | 4 | 0.014629231 |
| MF | GO:0042826 | histone deacetylase binding | 8 | 0.016553759 |
| MF | GO:0004520 | endodeoxyribonuclease activity | 4 | 0.028512883 |
| MF | GO:0008536 | Ran GTPase binding | 4 | 0.028512883 |
| MF | GO:0017056 | structural constituent of nuclear pore | 3 | 0.029344745 |
| MF | GO:0097472 | cyclin-dependent protein kinase activity | 3 | 0.035155375 |
| MF | GO:0016896 | exoribonuclease activity, producing 5'-phosphomonoesters | 4 | 0.038467319 |
| MF | GO:0004532 | exoribonuclease activity | 4 | 0.046199716 |
| MF | GO:0140098 | catalytic activity, acting on RNA | 13 | 0.046199716 |
| MF | GO:0042162 | telomeric DNA binding | 4 | 0.049548307 |
| MF | GO:0036002 | pre-mRNA binding | 5 | 0.049548307 |
